# Using Genome-Wide Association Studies to Reveal DArTseq and SNP Loci Associated with Agronomic Traits and Yield in Maize

**DOI:** 10.3390/cimb47121008

**Published:** 2025-11-30

**Authors:** Maciej Lenort, Agnieszka Tomkowiak, Jan Bocianowski, Roksana Bobrowska, Danuta Kurasiak-Popowska, Sylwia Mikołajczyk, Tomasz Kosiada, Dorota Weigt, Przemysław Gawrysiak

**Affiliations:** 1 Department of Genetics and Plant Breeding, Poznań University of Life Sciences, Dojazd 11, 60-632 Poznań, Poland; maciej.lenort@up.poznan.pl (M.L.); roksana.bobrowska@up.poznan.pl (R.B.); danuta.kurasiak-popowska@up.poznan.pl (D.K.-P.); sylwia.mikolajczyk@up.poznan.pl (S.M.); dorota.weigt@up.poznan.pl (D.W.); 2 Department of Mathematical and Statistical Methods, Poznań University of Life Sciences, Wojska Polskiego 28, 60-637 Poznań, Poland; 3 Department of Phytopathology, Seed Science and Technology, Faculty of Agronomy, Horticulture and Bioengineering, Poznań University of Life Sciences, Dąbrowskiego 159, 60-594 Poznań, Poland; tomasz.kosiada@up.poznan.pl; 4 Institute of Environmental Biology, Faculty of Biology, Adam Mickiewicz University in Poznań, Uni-wersytetu Poznańskiego 6, 61-614 Poznań, Poland; p.gawrysiak98@gmail.com

**Keywords:** *Zea mays*, yield, association mapping, next-generation sequencing (NGS), candidate genes

## Abstract

Next-generation sequencing (NGS) has revolutionized genetic research, enabling the massive, rapid, and relatively inexpensive analysis of the genomes, transcriptomes, and epigenomes of various organisms, including maize. Therefore, this paper uses NGS, association mapping, and physical mapping to identify candidate genes associated with yield structure traits and yield in maize (*Zea mays* L.). Furthermore, expression analysis of selected candidate genes was performed to confirm their contribution to yield formation. The plant material used for the study was 186 F1 hybrids and 20 reference genotypes (high-yielding and low-yielding). Field experiments were conducted simultaneously in two locations (in Smolice and Kobierzyce). NGS yielded a total of 45,876 molecular markers (24,437 SilicoDArT markers and 21,439 SNP markers) relevant to yield and crop structure. The largest number of markers in both localities (Smolice and Kobierzyce) was related to: the number of grain rows (6960), dry matter content after harvest (6616), the number of grains in a row (6721), mass of grain from the cob (6616), and cob length (6564). The smallest number of markers in both localities was related to yield (t ha^−1^) (1114) and yield from the plot (1237). To narrow down the number of markers for physical mapping, ten were selected from all the significant ones associated with the same traits in both localities (Kobierzyce and Smolice). Significant markers included eight silicoDArT markers (459199, 2447305, 4768759, 4579916, 4764335, 2448946, 2492509, 4774802) and two SNP markers (9692004, 5587791). These markers were used for physical mapping. These markers are located on chromosomes 7, 8, and 10. Some of these markers are located at a considerable distance from characterized genes or within uncharacterized genes. Two markers caught our attention: SNP 5587791 and silicoDArT 4774802. The first one is located on chromosome 8 inside exon 5 of the LOC100383455 U-box domain-containing protein 7 gene, the second marker is also located on chromosome 8 near (300 bp) the LOC103635953 putative WUSCHEL-related homeobox 2 protein gene. Our own research and literature reports indicate the usefulness of next-generation sequencing, association mapping, and physical mapping for identifying candidate genes associated with economically important traits in maize. Furthermore, two genes characterized in detail in the publication, LOC100383455 U-box domain-containing protein 7 gene and LOC103635953 putative WUSCHEL-related homeobox 2 protein gene, may be involved in processes related to maize yield.

## 1. Introduction

Maize (*Zea mays*) is the second most economically important crop after wheat. Globally, the area cultivated for grain maize is 197 million hectares, compared to 216 million hectares for wheat and 165 million hectares for rice [1]. Globally, annual maize grain production currently reaches 1137 million tons, significantly exceeding rice and wheat production. Over the past 25 years, maize production has more than doubled, reflecting both significant yield increases and expansion into increasingly wider areas. Of the three cereals, maize yields increased by almost 2 tons during this period. Average grain maize yields in Europe are approximately 7–10 t/ha. In some countries with favorable climatic conditions and high-quality soil, yields can exceed 12 t/ha (e.g., France, the Netherlands, Germany). If current trends continue and the stagnation in wheat area continues, maize has the potential to become the most widely cultivated crop by 2030 [2].

Despite good forecasts regarding the increase in corn yields, ongoing climate change may pose a significant threat. As stated by the Intergovernmental Panel on Climate Change (IPCC), heat waves are on the rise, growing more frequent and intense, bringing substantial dangers to human well-being, food safety, and natural surroundings. These extraordinary episodes of high temperatures are causing disruptions in everyday existence and applying pressure on worldwide farming output. Climbing temperatures and the connected heat strain are vital factors that influence harvest production, encompassing essential crops such as maize [3].

In the era of rapid development of molecular techniques, further increases in maize yields will be possible thanks to biological progress, which can be defined as an ecological approach to the intensification of agricultural production based on the genetic improvement of plants. These plants are becoming more efficient in the use of natural resources and industrial inputs [4]. Literature data clearly indicate that since 2010, genetic progress in maize breeding has significantly contributed to increased yields, improved plant resistance, and thus increased farmer incomes [5]. The search for new genes of economic importance is a key challenge for modern plant breeding [6,7,8]. Modern maize breeding relies on a wide range of molecular genetic research techniques. The development of modern biotechnological tools has significantly accelerated and streamlined selection processes and enabled the identification of genetic modifications in maize varieties, which translates into increased breeding efficiency and improved plant functional traits. Molecular techniques such as genomic selection, CRISPR/Cas9 gene editing, and genome sequencing have become widely used in research on the genetic basis of traits such as disease resistance, abiotic stress resistance, herbicide tolerance, and yield [9,10]. This offers not only attractive prospects for biological progress but also new opportunities for the use of maize and other crop plants [11].

Next-Generation Sequencing (NGS) has revolutionized genomic biology and medicine by enabling massive, parallel reads of millions of DNA and RNA fragments in a short time and at a lower cost than classical Sanger sequencing [12]. In recent years, NGS developments have focused on increasing read length, improving database quality, reducing bias, and integrating with single-cell and epigenomic analyses [13,14]. Short-read technologies (Illumina) still dominate in terms of accuracy and price, but platforms such as Pacific Biosciences (PacBio) and Oxford Nanopore Technologies (ONT) are gaining importance due to their ability to read long DNA fragments, which facilitates de novo genome assembly, detection of genomic structures, and analysis of transcript isoforms [15,16].

In our research, we used DArTseq technology. It relies on restrictive DNA cleavage to prepare libraries of fragments and then sequence them using NGS platforms. This enables rapid and relatively inexpensive genotyping of large numbers of samples, which is particularly important in studies of genetic variation, genetic diversity, and plant and animal selection [17]. A significant advantage of DArTseq technology is its ability to analyze samples with low DNA quality, which provides significant convenience in practical applications, such as ecological studies or when access to fresh material is limited. Its multifaceted applications and the ability to integrate with other genetic technologies make DArTseq a key tool in modern genomics [18,19,20].

The advent of new genome sequencing technologies, combined with new computational methods, has led to the sequencing of the maize reference genome. The extensive genotypic data obtained from NGS can be used for association mapping. Genome-wide association studies (GWAS) have become a powerful tool for analyzing genetic variation and identifying relationships between traits and their underlying genetic variation, leveraging historical recombination events [21]. Association mapping involves searching for genotype-phenotype correlations in unrelated individuals using dedicated statistical methods [22,23,24]. This approach enables the generation of high-quality markers for marker-assisted selection (MAS). Functional markers, closely linked to a trait, reflect gene polymorphisms that directly influence phenotypic variation. Association mapping offers the potential to find specific markers across a wide range of genetic resources. The potential of association mapping stems from the ability to achieve higher resolution by utilizing a larger number of recombination events in the developmental history of germplasm [25]. Therefore, association mapping has emerged as a promising approach compared to traditional mapping. Initially, association mapping in maize [26] did not account for population structure, a finding that was corrected by Pritchard in 2001 [27], who incorporated this structure into his maize studies.

In recent years, maize breeding worldwide has benefited from the use of useful molecular markers, significantly improving yields in both the United States and other countries, offering enormous potential for increasing the productivity and value of maize germplasm [28,29]. Maize, like barley and rice, is one of the most genetically characterized cereal species, with over 32,000 genes distributed across ten chromosomes and a genome size of 2.3 Gbp. A hallmark of the maize genome is its high level of polymorphism. Many loci contain multiple active alleles, and the frequency of repeat DNA sequences, including retrotransposons and transposons, is approximately 58%. Genic regions constitute only 7.5% of the entire maize genome [30].

Over the past two decades, many researchers [31,32,33] have used molecular biology methods to detect and localize loci determining grain yield and yield structural traits in maize. In the context of time- and resource-saving molecular marker-based selection, there is a constant search for new markers related to yield and its components. Studies presented by various authors suggest that quantitative trait loci (QTLs) related to grain yield and its components are scattered throughout the genome. Prasanna et al. [34] showed that the highest number of QTLs related to grain yield in maize are located on chromosomes 1S, 1L, 2S, 5S, 6L, and 8L. Similar results were obtained by Beavis et al. [35], who also analyzed hectolitre weight of grain, locating QTL regions on chromosomes 1S, 2S, 3S, and 5S. Extensive studies by Ribaut et al. [36] and Veldboom et al. [37] showed that QTL regions related to the number of kernel rows are located on chromosomes 1L, 4L, 5L, and 9S, while QTLs related to the number of cobs per plant are located on chromosomes 1S, 1L, 3S, 3L, 6L, and 8L. They also determined the location of QTLs for cob length (chromosomes 1S, 1L, 3S, 3L, 5S, 6L, 8L) and cob diameter (chromosomes 1L, 2L, 4L, 7L, 8L). Studies on the identification of QTLs for yield structure traits were also conducted by Austin et al. [38] and Melchinger et al. [39].

Intensive molecular analyses in maize focus not only on identifying new markers and QTL regions but also on finding methods for selecting parental components for heterosis crosses [40]. It is well known that breeding success relies on access to starting materials with high genetic diversity, as well-defined and classified plant material reduces the costs of hybrid breeding. Plant materials can be classified into heterotic groups according to various criteria, such as genetic origin (pedigree), results of diallelic crosses, and geographic origin. Unfortunately, the presented classification criteria have certain limitations. Diallelic crosses provide a wealth of information about the available material, but these methods are often expensive. Inferring genetic diversity based on geographic origin is also problematic due to the international exchange of breeding materials. Furthermore, in the case of genetic origin, access to complete pedigree information can be limited. Therefore, in recent years, attempts have been made to select parental components for heterotic crosses based on the genetic similarity between the parental lines, determined using molecular markers [41,42].

Therefore, the primary goal of the study was to investigate the genetic mechanisms influencing maize yield. Using NGS and association mapping, the authors identified new silicoDArT and SNP markers associated with genes determining yield structure traits and yield in maize. Subsequently, physical mapping was used to determine the markers’ location and their association with nearby genes. Next, primers were designed for use in breeding programs to select high-yielding genotypes. Diagnostic procedures for identifying new molecular markers were optimized on high- and low-yielding materials. The expression levels of selected candidate genes were also determined.

## 2. Materials and Methods

### 2.1. Materials

Plant material consisted of 186 F1 hybrids sent for next-generation sequencing and 20 reference genotypes (ten high-yielding and ten low-yielding). Five reference genotypes were used for gene expression analyses (four high-yielding and one low-yielding as a negative control). Field observations of these five genotypes were conducted for three years (2018–2020). In total, 211 genotypes were used in the experiment. The F1 hybrids were obtained by crossing inbred lines, belonging to different origin groups, characterized by varied yields and yield structure traits. Some of the parent lines were flint grain lines of three different origins: F2 (a group related to the F2 line, bred at INRA in France from the Lcaune population), EP1 (a group related to the EP1 line, bred in Spain from the population derived from the Pyrenees), and German Flint. The other part of the parent lines were dent-type kernels derived from various origin groups in the United States: Iowa Stiff Stalk Synthetic (BSSS), Iowa Dent (ID), and Lancaster. Plant material came from two Polish breeding companies: Smolice Hodowla Roślin Sp. z o.o. and Małopolska Hodowla Roślin Sp. z o.o. from the IHAR Group. Information on the origin of the parental forms of the F1 hybrids was also obtained from the breeding companies.

### 2.2. Methods

#### 2.2.1. Phenotyping

##### Field and Phytotron Experiments

The experiment with 186 F1 hybrids and 20 reference genotypes (high-yielding and low-yielding) was conducted on plots measuring 10 m^2^, in a randomized complete block design, with three replicates, at two locations: Smolice 51°42′58.904″ N 17°13′29.13″ E and Kobierzyce 50°58′19.411″ N 16°55′47.323″ E. Throughout the entire growing season, field observations were made, including the degree of plant infestation by pests and diseases (fungi of the *Fusarium* genus). Immediately after harvest, biometric measurements were taken from the ears, such as: 1—ear length, 2—ear diameter, 3—kernel length, 4—kernel diameter, 5—the number of grain rows, 6—the number of grains per row, 7—kernel mass per ear, 8—thousand kernel weight, 9—yield per plot, 10—dry matter content after harvest, 11—yield (t/ha), The experiment with five reference genotypes was carried out in a controlled environment chamber under regulated conditions. The temperature was set at 21 °C during the day and 19 °C at night, with a 16-h photoperiod, and the relative humidity (RH) ranged from 65% to 75%. The light intensity (PPFD) was 700 µmol m^–2^ s^−1^. Four maize kernels were sown into pots with a diameter of 16 cm filled with soil from the field, with four replicates. The soil was maintained at approximately 70%. The conformity of the empirical distributions of four analyzed morphological pollen traits with the normal distribution was assessed using the Shapiro–Wilk *W*-test. Homogeneity of variances was evaluated using Bartlett’s test.

##### Meteorological Conditions During the 2021 and 2022 Growing Seasons

**Figure 1:**
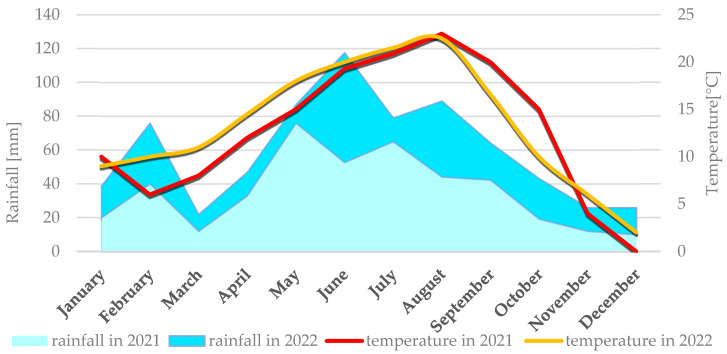
Average monthly temperature and average monthly precipitation in Smolice in 2021 and 2022.

**Figure 2:**
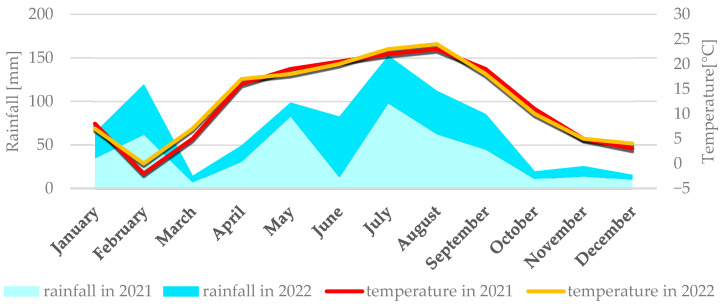
Average monthly temperature and average monthly precipitation in Kobierzyce in 2021 and 2022.

Average weather conditions for both locations in 2021 and 2022 are described simultaneously because they didn’t differ significantly. Weather conditions for each location are presented individually in Figure 1 (Smolice) and Figure 2 (Kobierzyce). During the 2021 growing season, weather conditions favored the growth and development of maize, although April frosts delayed sowing. May, a key month for this crop’s growth and development, was cool (average 12 °C) and wet, with total precipitation of 76 mm. In contrast, June and July 2021 were dry (52.7 mm and 65 mm, respectively) and relatively warm (average temperatures 19.3 °C in June and 20.9 °C in July). Such dry, warm weather limited the development of fungal diseases during that period. In August, the risk of *Fusarium* infection in maize increased, mainly due to high rainfall (140.1 mm) and temperatures around 17 °C. Dry, warm conditions in September (42.3 mm of rain) and October (19.2 mm) suppressed the development of fungal diseases, including *Fusarium* ear rot. As a result, all examined genotypes showed high resistance to these pathogens.

The 2022 season was also characterized by conditions favorable for maize growth. May was very warm and dry, with an average temperature of 15.2 °C and just 10.8 mm of precipitation. Low humidity and lack of rain intensified drought. The following months—June, July, and August—were above average in temperature: 18.3 °C, 21.7 °C, and 22.1 °C, respectively. June had the highest rainfall in that period (63.4 mm), while July (13.3 mm) and August (44.8 mm) were exceptionally dry. September was warm (average 16.7 °C) with light rainfall (22.5 mm), as was October (10.2 °C, 24.6 mm). Such dry, hot weather limited the development of fungal diseases throughout the growing season.

#### 2.2.2. Genotyping

##### DNA Isolation

DNA isolation was performed using a reagent kit from Promega GmbH; Gutenbergring 10; 69190 Waldorf, Germany (Maxwell^®^ RSC PureFood GMO and Authentication Kit). The concentration and purity of the isolated DNA samples were checked using a DeNovix DS-11 spectrophotometer (DeNovix Inc.; 3411 Silverside Rd; Hanby Building Wilmington, DE 19810 USA). The isolated DNA template was adjusted to a uniform concentration of 100 ng/μL by dilution with distilled water.

##### Associative Mapping Using GWAS Analysis

Associative mapping for yield and yield-structure traits was performed on 186 F1 maize hybrids using GWAS analysis [43]. Association mapping was conducted based on species mean trait values and the generated marker data, using a mixed linear model (MLM) approach. This model incorporated population structure inferred via eigenanalysis and modeled as random effects [44,45]. All statistical analyses and result visualizations were carried out using Genstat 23.1 software [46], specifically employing the QSASSOCIATION procedure. This procedure implements a mixed-model marker–trait association analysis (also known as linkage disequilibrium mapping) for data obtained from a single-trait trial. To control for false-positive associations due to population structure and relatedness, the model included a genetic relatedness correction. The RELATIONSHIPMODEL = eigenanalysis option was used, which identifies major principal components from the marker matrix to account for population stratification [47]. The scores of the significant principal components were incorporated as covariates in the MLM, providing an approximation of the genetic variance–covariance structure via a kinship matrix. The statistical significance of marker–trait associations was evaluated using *p*-values adjusted for multiple testing using the Benjamini–Hochberg false discovery rate correction method. This mapping was based on results obtained from genotyping and phenotyping. Genotyping was performed using DArTseq technology, based on next-generation sequencing. Isolated DNA from the tested maize plants, 25 µL at a concentration of 100 ng per genotype, was sent to two 96-well Eppendorf plates for analysis, the aim of which was to identify silicoDArT polymorphisms and SNPs. Analyses were performed at Diversity Arrays Technology, University of Canberra, Australia. After DNA isolation, the next step was to digest the genomic DNA with restriction enzymes such as Ape KI, Pst I, and Msp I to reduce genome complexity. This allowed for the creation of a homogeneous library and enabled the detection of most fragments associated with the rare-cutting enzyme. The restriction enzymes used are sensitive to methylation, allowing for the filtering out of non-coding regions and methylated repetitive sequences, such as mobile elements. Genomic DNA fragments cut by restriction enzymes were ligated to adaptors. The adaptors contained a barcode, ensuring the origin of each sample was precisely defined and the identifiers met appropriate criteria. The resulting PCR products were analyzed for size and constituted a genomic library, which was then sequenced using the leading NGS platform, Illumina, according to the methodology described in detail on the Diversity Arrays Technology website: URL (https://www.diversityarrays.com/technology-and-resources/dartseq/, accessed on 16 November 2020). Phenotypic data comprised biometric measurements: 1—cob length, 2—cob diameter, 3—core length, 4—core diameter, 5—the number of rows of grain, 6—the number of grains in a row, 7—mass of grain from the cob, 8—weight of one thousand grains, 9—yield from the plot, 10—dry matter content after harvest, 11—yield (t ha^−1^). The field experiment was conducted in 2021–2022 on 10 m^2^ plots, in a complete randomized block design, with three replications, in two locations (Kobierzyce and Smolice). All environments were included in the GWAS study. Significance was set at the level of 0.001. The results are presented in Manhattan plots.

##### Physical Mapping

The sequences of silicoDArT markers and SNPs, selected based on GWAS analysis, were subjected to BLAST (Basic Local Alignment Search Tool) —version BLAST+ 2.17.0 searches, which involve searching databases to find sequences with a high degree of similarity to the selected markers sequence. This analysis was performed using the URGI (Unité de Recherche Génomique Info) platform, utilizing the fully sequenced maize genome. The URGI tool allowed for the determination of the chromosomal locations of the searched sequences and their physical positions on the chromosome. To identify the most probable region containing the most similar sequences, the combined probability was calculated based on the E-value for each chromosome. All genes within this identified region were further analyzed.

##### Functional Analysis of Gene Sequences

The functional analysis was performed using the Blast2GO software URL (https://www.blast2go.com/, accessed on 20 April 2023). Sequences of all genes located within the chromosomal regions identified based on BLAST analysis in the URGI service were analyzed. The aim was to obtain information on the biological function of the gene sequences located in the specified chromosomal regions.

#### 2.2.3. Identification of Selected SNP Polymorphisms and SilicoDArT on Agarose Gels

##### Designing Primers for Identified Polymorphisms of SilicoDArT and SNP Associated with Yield and Yield Structure Traits

For each marker, pairs of PCR primers were designed, with one primer targeting the identified Indel or SNP, and the other being complementary to the sequence adjacent to the analyzed marker. In the case of SNP identification, polymorphic nucleotide positions were located at the 3′ end of the appropriate primer. Additionally, to increase the performance difference in amplification initiation between marker and non-marker sequences containing different SNPs, the penultimate nucleotide at the 3′ end of each SNP-containing primer was replaced with an appropriate non-complementary nucleotide.

##### Identification of Selected Molecular Markers Linked to Yield and Yield Structure Traits Using Polymerase Chain Reaction (PCR)

The identification of ten specific SilicoDArT and SNP markers linked to genes related to the characteristics of the crop structure and yield was conducted using PCR. A 25 µL reaction mix contained: 10× ThermoPol reaction buffer, 2.5 µL; 100 mM MgSO_4_, 1.5 µL; 10 mM Ultrapure dNTPs Mix, 0.5 µL; Deep Vent (exo) DNA Polymerase (New England Biolabs), 0.25 µL; nuclease-free water, 16.25 µL; 10 µM Forward primer (F), 1 µL; 10 µM Reverse primer (R), 2 µL; and DNA. PCR conditions for SNP identification were as follows: initial denaturation at 95 °C/5 min; denaturation at 95 °C/30 s; primer annealing at Ta/30 s (Table 1); extension at 72 °C/30 s; final denaturation at 72 °C/5 min; hold at 4 °C/∞; total number of cycles 40.

##### Identification of Selected SNP and SilicoDArT Polymorphisms on Agarose Gels

PCR products were separated in a 2% agarose gel with 1 µL of Midori Green stain, for 2 h at 100 V. To identify amplification products, the size marker O’RangeRuler 50 bp and 100 bp (Fermentas) was used. Visualization of DNA fragments was performed under UV light and documented using digital images with the BIORAD gel imaging and documentation system.

#### 2.2.4. Analysis of the Expression of Selected Genes Linked to Yield-Related Traits and Maize Yield

##### mRNA Isolation

RNA was isolated from five reference genotypes (five genotypes × three biological replicates × five time points, a total of 75 plants) subjected to gene expression analysis, using reagents from Promega GmbH; Gutenbergring 10; 69190 Waldorf; Germany (Maxwell^®^ RSC Plant RNA Kit). RNA was isolated from maize leaves in the waxy ripeness phase (BBCH 85). For the expression analyses, plant samples were collected before inoculation and at 6 h, 12 h, 24 h, and 72 h post-inoculation. A total of 75 samples were analyzed. Genotypes marked with the letter S came from the Smolice HR, while those marked with the letter K came from the Małopolska HR. These genotypes were selected based on field observations conducted over three consecutive years (2018–2020).

##### cDNA Synthesis

cDNA synthesis was performed using the iScript cDNA Synthesis Kit from BIO-RAD, 1000 Alfred Nobel Dr, Hercules, CA 94547, USA. The synthesis was conducted for 75 samples of previously isolated mRNA. The reaction mixture included mRNA, water, and RT Supermix. The prepared samples were incubated according to the following protocol:
Preparation5 min at 25 °CReverse transcription20 min at 46 °CInactivation1 min at 95 °C

##### Expression Analysis Using RT-qPCR

The sequences of the studied genes were used to design the primers for the qPCR: LOC100383455 (encoding *U*-box domain-containing protein 7) and LOC103635953 (putative WUSCHEL-related homeobox 2 protein). For each gene (for each pair of primers), a PCR with a temperature gradient was performed to determine the annealing temperature. The gradient was set in the temperature range from 54 °C to 58 °C. The PCR products were separated on a 1.5% High Resolution Plus agarose gel (Bioshop, Canada Inc., Burlington, ON, Canada) in 1× TBE buffer (Bioshop, Canada Inc., Burlington, ON, Canada), using Midori Green Direct DNA Stain (Nippon Genetics Europe, Mariaweilerstraße 28 a, 52349 Düren, Germany). The electrophoresis results were visualized on a Molecular Imager Gel Doc™ XR UV system with Bio Image™ software version 5.2.1 (Bio-Rad Laboratories, Inc., Hercules, CA, USA). After obtaining a specific amplification product on a 1.5% agarose gel, the annealing temperature for qRT-PCR was set to 55 °C. Two reference genes (β-tubulin and cyclophilin) were selected. qRT-PCR analysis was performed using iTaq Universal SYBR Green Supermix and the CFX Connect Real-Time PCR Detection System (Bio-Rad Laboratories, Inc., Hercules, CA, USA). Each qRT-PCR experiment consisted of three biological and three technical replicates, the results of which were calculated and averaged. During each individual expression analysis, a negative control, NTC (No Template Control), was run on a 96-well plate in three technical replicates. The following temperature profile was used for the qPCR: initial denaturation for 3 min at 95 °C, and then 40 cycles of denaturation for 10 s at 95 °C, primer annealing for 30 s at 55 °C (fluorescence measurement). Melting degree (melting curve): The melting temperature range was 65–90 °C; every 5 s, the temperature was increased by 0.5 °C and measurement was performed. The results of standard curves for qRT-PCR efficiency (%E) and coefficient of determination (*R*^2^ values) were included in the calculation of expression results. Normalized expression was calculated using CFX Maestro version 4.0 software and the Gene Study tool (Bio-Rad Laboratories, Inc., Hercules, CA, USA), which allowed comparison of the expression of both genes for biological replicates of each genotype at selected time points. Primer sequences for the analyzed genes are shown below:

LOC100383455 (encoding U-box domain-containing protein 7)
5′TCTGACTGGCTCTGAAGACG3′
3′TACCTGAGCTCCAACATCCAG5′
Product length 223 bp
LOC103635953 (putative WUSCHEL-related homeobox 2 protein)
5′CGGCGTACGGCTACTACTAC3′
3′GCTGCCACCCGTCGTG5;
Product length 128 bp

The Kolmogorov–Smirnov test was used to test the null hypothesis that the empirical distribution of normalized expression data for a specific gene and line complied with the normal distribution. This test was applied to all gene × line combinations. To assess the homogeneity of variances, the Brown–Forsythe variant of Levene’s test was calculated (passed for all tested time point × gene × line combinations). For comparisons of time measurements, a repeated measures ANOVA was performed, and then *t*-tests were performed between the lines per time point to analyze the time and line interactions at the significance level of *p* < 0.05. The *p*-values were adjusted using Benjamini–Hochberg correction for multiple testing. All these analyses were conducted using the Genstat v.23 statistical software package [46].

##### Reference Gene Expression Analysis

Two reference genes were selected: β-tubulin and cyclophilin, based on the highest reaction efficiency (%E) values and determination coefficient (R^2^ > 0.997 for β-tubulin and R^2^ > 0.996 for cyclophilin). The applied temperature gradient allowed establishing 60 °C as the optimal primer annealing temperature during RT-qPCR for all analyzed genes. The results of the reference gene analysis were compared with the expression of candidate genes using the Gene Study tool (CFX Maestro, Bio-Rad Laboratories, Inc., Hercules, CA, USA). Primer sequences for the selected reference genes are shown below:

β-tubulin
5′CTACCTCACGGCATCTGCTATGT3′
3′AACACGAATCAAGCAGAG5′
Cyclophilin
5′CTGAGTGGTGGTCTTAGT3′
3′GTCACACACACTCGACTTCACG5′

## 3. Results

### 3.1. Field Experiment

**Figure 3:**
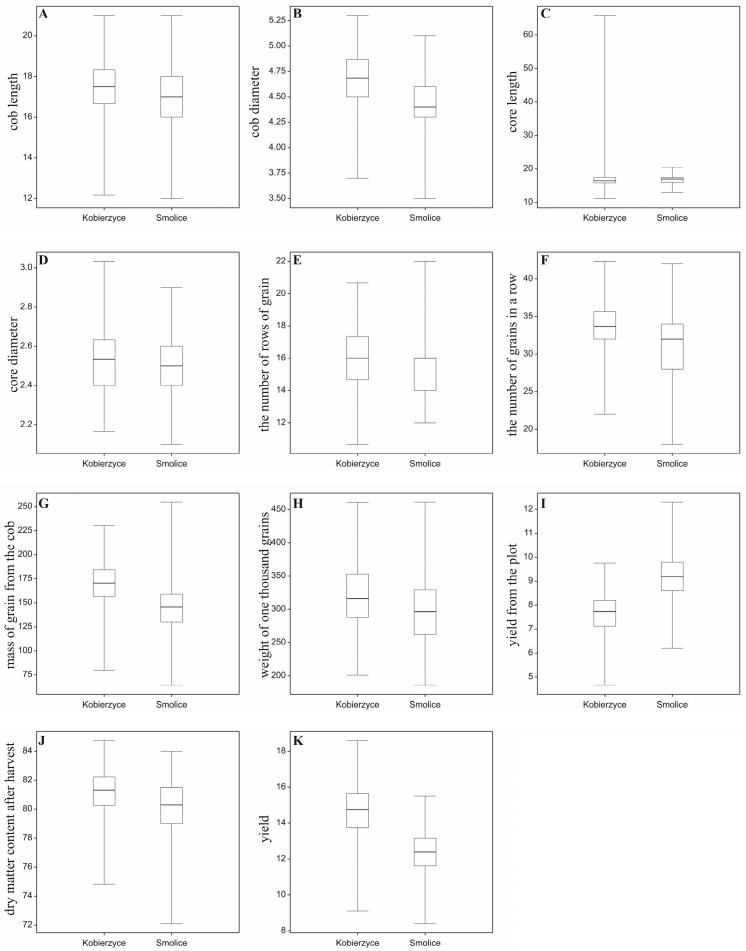
Boxplots illustrating the distribution of the analyzed variables for the studied traits. (**A**) cob length, (**B**) cob diameter, (**C**) core length, (**D**) core diameter, (**E**) the number of rows, of grain, (**F**) the number of grains in a row, (**G**) mass of grain from the cob, (**H**) weight of one thousand grains, (**I**) yield from the plot, (**J**) dry matter content after harvest, (**K**) yield.

A field experiment with maize hybrids was established in 2022 and 2023 at two locations: Smolice and Kobierzyce. The Shapiro–Wilk *W*-test for normality ranged from 0.969 (*p* = 0.099 for the number of grains in a row) to 0.987 (*p* = 0.773 for yield). The Bartlett χ^2^ test ranged from 9.66 (*p* = 0.855 for core diameter) to 17.73 (*p* = 0.281 for yield). To examine the distribution of all analyzed variables at both locations, boxplots were prepared (Figure 3). The plots illustrate the ranges in which trait values are concentrated; for example, cob length for most genotypes at both locations (Smolice, Kobierzyce) falls between 17 and 19 cm, cob diameter for most genotypes in Smolice is 4.65–4.70 cm, and in Kobierzyce 4.55–4.80 cm (Figure 3). As shown in the attached plots, the distributions of several variables differed between locations: core diameter, the number of rows of grain, the number of grains in a row, mass of grain from the cob, yield from the plot, and yield (t ha^−1^) (Figure 3).

Analysis of variance was conducted on the observed traits across genotypes. Significant differences were found for all traits between genotypes. Additionally, the analysis revealed statistically significant differences for all observed traits between the locations where the field experiment was conducted (Table 1). In the Supplementary Materials, the data in Tables S1 and S2 present the mean values of the observed traits for individual hybrids.

**Figure 4:**
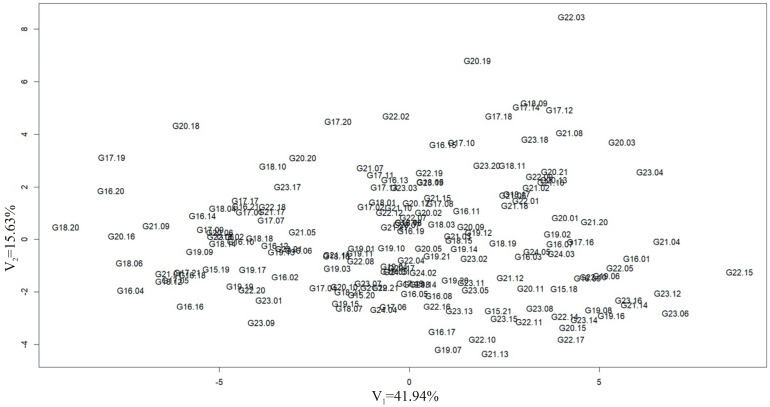
Distribution of corn hybrids in the arrangement of the first two canonical variables.

To determine the relationships between groups of variables in the dataset, i.e., observations of yield structure traits and yield, in both locations, a multivariate technique, i.e., canonical variate analysis, was used. All observed traits were characterized by a normal distribution. It can be seen that some hybrid forms deviate from the mean trait values for the analyzed population (G22.03, G20.19, G20.18, G17.19, G16.20, G18.20, and G22.15) (Figure 4).

**Figure 5:**
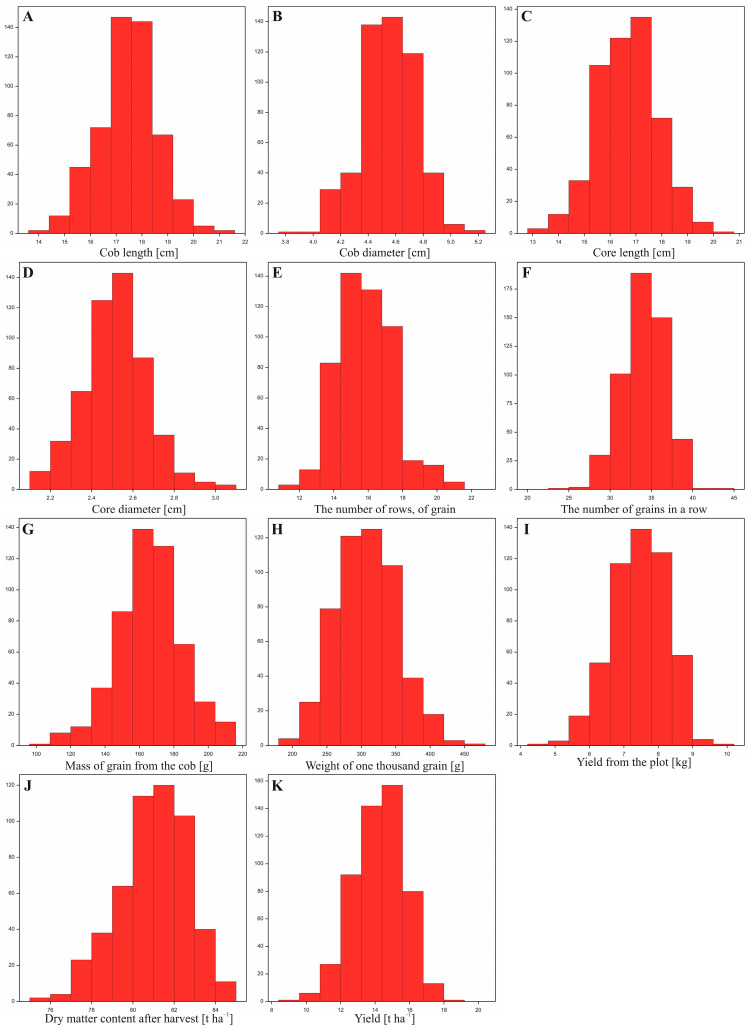
Distribution of individual traits analyzed in the villages of Kobierzyce and Smolice. (**A**)—cob length, (**B**)—cob diameter, (**C**)—core length, (**D**)—core diameter, (**E**)—the number of rows, of grain, (**F**)—the number of grains in a row, (**G**)—mass of grain from the cob, (**H**)—weight of one thousand grains, (**I**)—yield from the plot, (**J**)—dry matter content after harvest, (**K**)—yield.

Using data from a field experiment conducted in both localities (Smolice and Kobierzyce), the distribution of the analyzed traits within the studied population was examined. Statistical analyses showed that for all analyzed traits, the distribution of traits assumed a symmetrical bell shape, the mean and median were equal (both measures were located in the middle of the distribution). 68% of the data were within one standard deviation of the mean, 95% of the data were within two standard deviations of the mean, and 99% of the data were within three standard deviations of the mean. The presented histograms show the frequency distribution of observations of individual features for both locations combined. All of them indicate that the empirical distributions are consistent with the normal distribution, which was confirmed by appropriate statistical tests (Figure 5).

**Figure 6:**
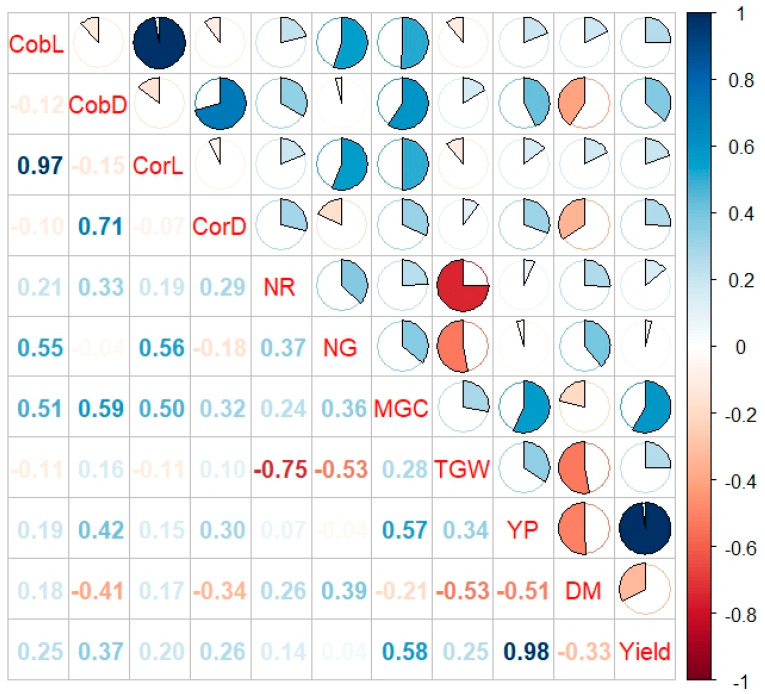
Correlations between the analyzed yield structure features in Smolice and Kobierzyce. CobL—cob length, CobD—cob diameter, CorL—core length, CorD—core diameter, NR—the number of rows of grain, NG—the number of grains in a row, MGC—mass of grain from the cob, TGW—weight of one thousand grains, YP—yield from the plot, DM—dry matter content after harvest, yield (t ha^−1^).

Correlations between the observed traits were analyzed. The strongest positive correlations were found between yield from the plot and yield (0.98), cob length and core length (0.97), and cob diameter and core diameter (0.71) (Figure 6).

### 3.2. Genotyping and Association Mapping

**Figure 7:**
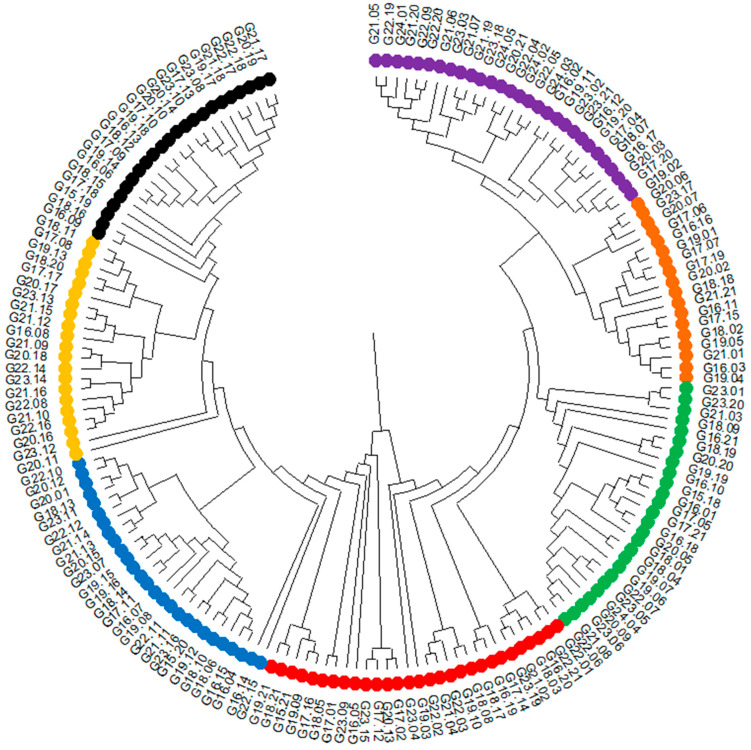
Dendrogram showing the genetic similarity between the analyzed genotypes. Different similarity groups are marked with different colors.

The raw next-generation sequencing results are deposited on the Diversity Arrays Technology website at URL https://www.diversityarrays.com/, accessed on 15 March 2022, under the user account (Agnieszka Tomkowiak). The results were used to conduct association mapping and statistical analyses. Based on the identified SNP and SilicoDArT molecular markers, a dendrogram of genetic similarity was generated between the 186 analyzed hybrids (Figure 7). Based on the genetic similarity assessment, hybrids were selected for subsequent crosses. The dendrogram distinguishes seven distinct similarity groups. The first group includes 23 genotypes from G21.17 to G16.09, and the second group includes 21 genotypes from G18.11 to G20.11. The third group consists of 27 genotypes from G22.10 to G22.15, and the fourth group consists of 29 genotypes from G19.21 to G22.01. The fifth group consists of 26 genotypes from G22.06 to G23.01, and the sixth group consists of 18 genotypes from G19.04 to G20.06. The seventh and final group consists of 29 genotypes from G19.02 to G21.05 (Figure 7).

**Figure 8:**
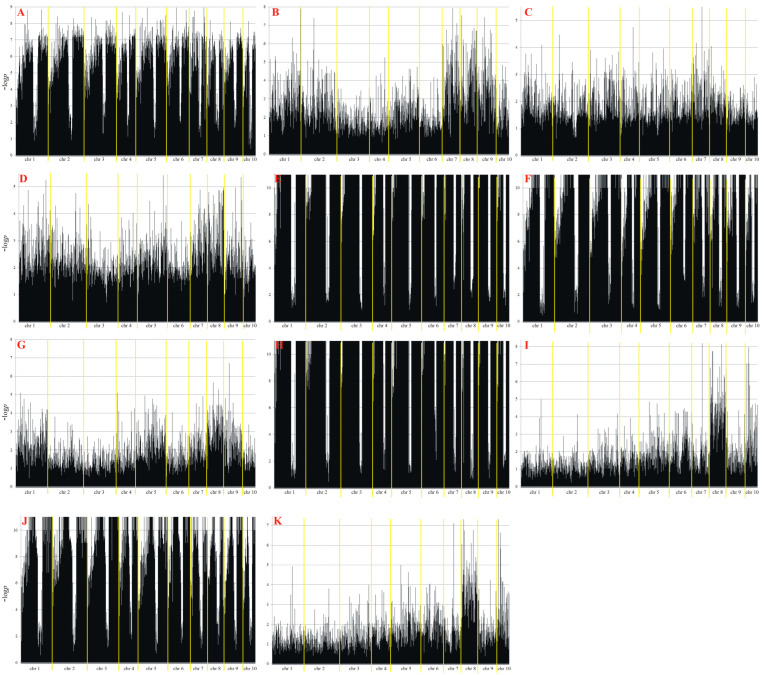
Manhattan plot for observed traits ((**A**)—cob length, (**B**)—cob diameter, (**C**)—core length, (**D**)—core diameter, (**E**)—the number of rows, of grain, (**F**)—the number of grains in a row, (**G**)—mass of grain from the cob, (**H**)—weight of one thousand grains, (**I**)—yield from the plot, (**J**)—dry matter content after harvest, (**K**)—yield). The statistical significance of marker–trait associations was evaluated using *p*-values adjusted for multiple testing using the Benjamini–Hochberg false discovery rate correction method.

Next-generation sequencing yielded a total of 45,876 SilicoDArT and SNP molecular markers relevant to yield and yield structure traits. A MAF > 0.25 and a missing observation rate < 10% were used to determine the suitability of the identified markers (Table 2). The largest number of markers in both localities (Smolice and Kobierzyce) was related to: the number of rows of grain (6960), dry matter content after harvest (6616), the number of grains in a row (6721), mass of grain from the cob (6616) and cob length (6564). The smallest number of markers in both localities was related to yield (t ha^−1^) (1114) and yield from the plot (1237). In order to narrow down the number of markers for physical mapping, 10 were selected from all the significant ones, which were associated with the same traits in both localities (Kobierzyce and Smolice) (Table 3, Figure 8).

### 3.3. Physical Mapping

From a total of 45,876 SilicoDArT and SNP markers, ten markers were selected that were most strongly associated (highest LOD values) with all analyzed traits combined (yield structure traits and yield) in both localities (Kobierzyce and Smolice) (Table 3). An attempt was made to determine the location of the selected markers. The next step was to design primers that were used to identify the ten selected markers (Table 4).

### 3.4. Verification of Selected Molecular Markers Using Designed Primers

**Figure 9:**
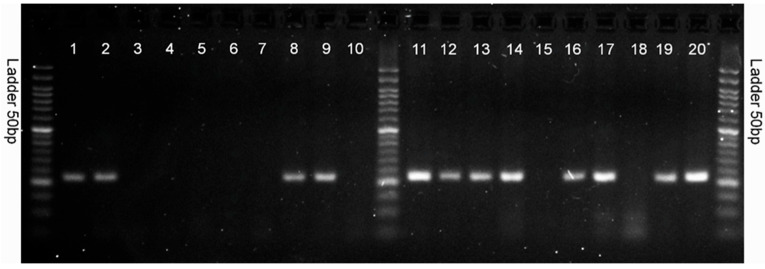
Electrophoresis image showing PCR products using primer pair: 5587791_SNP_F and 5587791_SNP_R. From numbers 1 to 10, these are high-yielding genotypes (1–5 from HR Smolice and 6–10 from Małopolska HR); from numbers 11 to 20, these are low-yielding genotypes (11–15 from HR Smolice and 16–20 from Małopolska HR).

Polymerase chain reaction (PCR) was used to identify the selected 10 markers. The markers were tested on 20 high- and low-yielding reference genotypes from HR Smolice (10 genotypes) and Małopolska HR (10 genotypes). The genotypes were presented on agarose gels in the following order: from number 1 to 10, these are high-yielding genotypes (1–5 from HR Smolice and 6–10 from Małopolska HR); from number 11 to 20, these are low-yielding genotypes (11–15 from HR Smolice and 16–20 from Małopolska HR) (Figure 9). Of the 10 markers, 1 SNP (5587791) differentiated the analyzed high- and low-yielding genotypes (Figure 9). This marker is located in exon 5 of LOC100383455 U-box domain-containing protein 7. In the case of this marker, a differentiating amplification product of 282 bp appeared in all low-yielding genotypes except 15 and 18, whereas in the case of high-yielding genotypes, it appeared only in genotypes 1, 2, 8 and 9 (Figure 9). Since yield is a polygenic trait, a larger pool of differentiating markers should be used to group genotypes into high- and low-yielding ones. The second marker coupled to the putative WUSCHEL-related homeobox 2 protein gene did not differentiate high- and low-yielding genotypes on agarose gels because amplification products of the desired size did not appear on the agarose gel. Nonspecific products were observed on the gel for all analyzed genotypes.

### 3.5. Analysis of the Expression Level of Selected Candidate Genes Using qPCR

After detailed analysis of the results, it was decided to examine the expression of two candidate genes. The location of the identified marker was the criterion for gene selection. The two selected genes were: LOC100383455 (encoding U-box domain-containing protein 7 and LOC103635953 (putative WUSCHEL-related homeobox 2 protein). On chromosome 8, in exon 5 of the former gene, a specific marker, SNP 5587791, is located. Also, on chromosome 8, near the latter gene (300 bp), is a specific marker, silicoDArT 4774802. Expression levels were studied in five genotypes: four high-yielding (S1, S2, K1, K2) and one low-yielding (S3). RNA was isolated from maize leaves in the waxy ripeness phase (BBCH 85).

**Figure 10:**
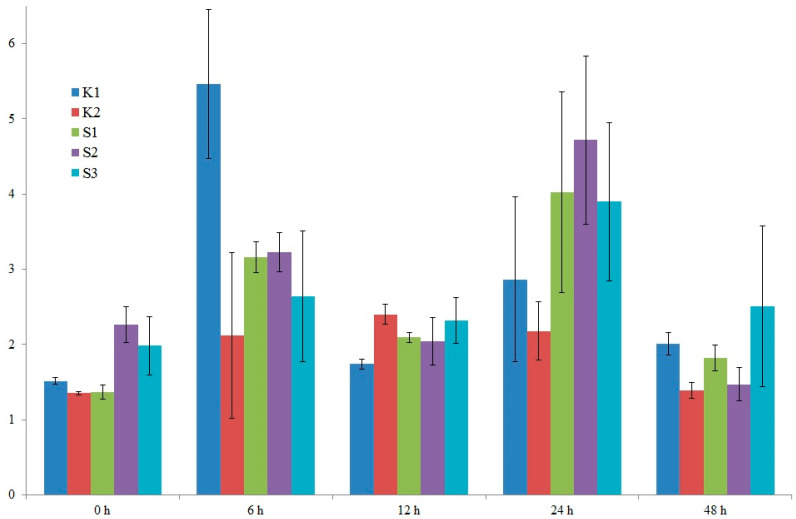
Graph showing changes in the expression level of the LOC100383455 U-box domain-containing protein 7 gene from 0 to 48 h. Five genotypes were analyzed: four high-yielding (S1, S2, K1, K2) and one low-yielding (S3).

**Figure 11:**
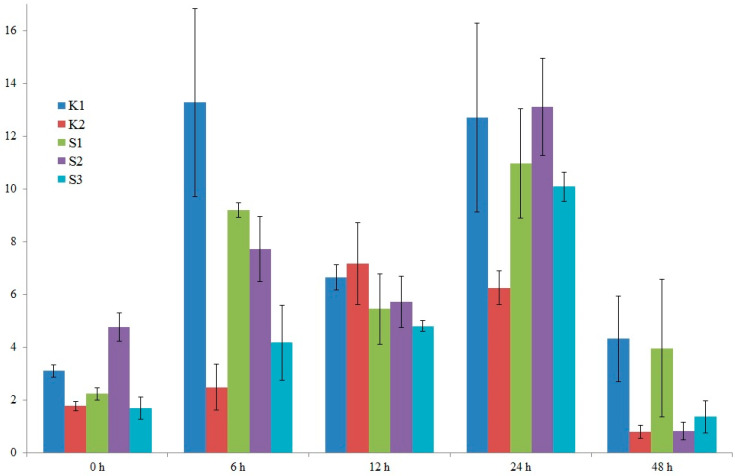
Graph showing changes in the expression level of the LOC103635953 putative WUSCHEL-related homeobox 2 protein gene from 0 to 48 h. Five genotypes were analyzed: four high-yielding (S1, S2, K1, K2) and one low-yielding (S3).

In the case of the LOC100383455 U-box domain-containing protein 7 gene, we observe variable expression levels from 0 to 48 h. It can also be seen that the high-yielding K1 genotype exhibits the greatest fluctuations in this gene’s expression level, especially after 6 h (Figure 10). A similar situation is observed for the LOC103635953 putative WUSCHEL-related homeobox 2 gene. Here, too, in the high-yielding K1 genotype, we observe the greatest fluctuations in this gene’s expression level (Figure 11). Differences in the expression profiles of yield-related genes between cultivars result from a combination of genetic, molecular, and contextual factors. In the case of the low-yielding S3 genotype, we also observe variable expression profiles of both analyzed genes. The expression levels of both genes in the S3 genotype are lower than in the K1 genotype but comparable to the K2, S1, and S2 genotypes. In genotypes with extreme traits, the genes responsible for these traits may exhibit similar expression for several reasons. For example, the extreme phenotypes may share similar epigenetic modifications (e.g., DNA methylation, histone modifications) that determine the activity of the same genes, leading to their similar expression levels. The organism may also activate regulatory mechanisms that equalize gene expression in the extreme phenotypes to maintain homeostasis and stability of biological functions.

## 4. Discussion

Many research centers around the world are conducting intensive research on the structure and function of the maize genome, utilizing modern methods of biotechnology and molecular biology. As a result of comprehensive breeding experiments, phenotypic observations, and genetic analyses, numerous QTLs associated with specific quantitative traits, such as yield and yield structure traits, have been identified [48]. This publication presents research on the analysis of maize hybrids from both phenotypic and genotypic perspectives to identify molecular markers linked to genes determining yield and its components. In this publication, analysis of variance was conducted based on phenotypic observations (related to yield and yield structure traits). Analysis of variance revealed statistically significant variation for all studied traits between the locations where the field experiment was conducted (Table 1). Whereas canonical variate analysis showed that all traits were normally distributed (Figure 4). Currently, traditional breeding methods are insufficient; therefore, modern breeding programs utilize high-throughput techniques for analyzing crop genomes, including maize [49]. This genomics-oriented approach allows for the acquisition of information about coding regions, which provide information about protein (gene) structure, as well as about intergenic regions [50]. Progress in the development of high-throughput DNA sequencing methods has led to a new level of research in many plant species, including maize [51,52]. Since the genome of the first model plant was sequenced in 2000, sequences from over 100 other plant species have been recorded [53,54]. These studies enable the detection of SNPs and their correlations with specific traits, as well as genomic selection, which enables the monitoring of entire genome segments in recombinant breeding programs.

In recent years, many authors have attempted to identify molecular markers functionally linked to important maize traits. Bocianowski et al. [55] used NGS and association mapping to identify markers associated with heterosis effects in maize. Using the same methods, Sobiech et al. [56] identified markers associated with Fusarium resistance in maize plants. NGS technology is used to sequence genomes and transcriptomes, study protein–DNA/RNA interactions, assess methylation levels, discover novel DNA polymorphisms, and conduct metagenomic studies [57]. This technology allows for the analysis of different DNA fragments represented by multiple copies in a single reaction, library preparation, and subsequent collection of gigabases of genomic data from a single sequencing run [58,59]. This not only increases the number of samples analyzed but also enhances the reliability of the obtained sequencing results. This is particularly valuable when differences between specific genotypes are small. The costs and time required for sequencing, per unit of information obtained, are significantly lower compared to analyses performed using traditional capillary sequencers [60]. Another sequencing strategy, primarily used to study plant-environment interactions, is the use of NGS methods to characterize the plant transcriptome in various physiological states. Analysis of cDNA sequences provides information about sequences transcribed in specific tissues or organs, and despite certain limitations, these data are very useful for breeders [61,62]. Next-generation sequencing techniques also enable qualitative and quantitative analyses of genes expressed under different conditions, and the results of these analyses are used for association mapping [63,64].

This publication presents the usefulness of field, molecular, bioinformatic, and statistical analyses in identifying candidate genes linked to genes related to yield and yield structure traits. Furthermore, methods for identifying candidate genes that can be used to select genotypes with desirable traits are proposed. This will save the financial resources required to breed maize varieties using traditional methods. Next-generation sequencing yielded a total of 45,876 SilicoDArT and SNP molecular markers relevant to yield and crop structure. To narrow down the number of markers for physical mapping, 10 were selected from all the significant ones associated with the same traits in both localities (Kobierzyce and Smolice). Significant markers included eight silicoDArT markers (459199, 2447305, 4768759, 4579916, 4764335, 2448946, 2492509, 4774802) and two SNP markers (9692004, 5587791). These markers were used for physical mapping. These markers are located on chromosomes 7, 8, and 10 (Table 3). Some of these markers are located at a considerable distance from characterized genes or within uncharacterized genes. Two markers caught our attention: SNP 5587791 and silicoDArT 4774802. The first one is located on chromosome 8 inside exon 5 of the LOC100383455 U-box domain-containing protein 7 gene, and the second marker is also located on chromosome 8 near (300 bp) the LOC103635953 putative WUSCHEL-related homeobox 2 gene.

According to literature reports, the U-box domain-containing protein 7 gene in plants is often referred to as PUX7. It is a gene that encodes a protein involved in protein degradation pathways, specifically interacting with ubiquitin and the AAA-ATPase AtCDC48A. This function is mediated through its UBX domain, which acts as a bridge between CDC48 and ubiquitin. U-box proteins have been identified in various plants, and the number of genes varies depending on the species. The genomes of *Arabidopsis thaliana*, tomato, peach, grape, banana, rice, and soybean contain 60, 62, 54, 56, 91, 76, and 120 U-box genes, respectively [65,66,67,68,69,70]. Numerous studies have been conducted to investigate the distinct functions of *U*-box genes. The authors suggest that U-box proteins participate in cell death signaling [71], flowering [72], and stress resistance [73,74]. U-box 13/43/50/51 genes were also found to be highly expressed and predicted to play a role in tomato fruit development and ripening. Studies by Li et al. [75] suggest that *U*-box proteins significantly participate in the regulation of organ growth and development. All these results indicate a diverse, crucial role for *U*-box genes in various biological processes, including yield formation.

Regarding the second gene, wuschel-related homeobox (WOX) gene family belongs to the homeodomain (HD) superfamily, which encodes one of the largest groups of transcription factors containing 60–66 amino acids conserved DNA-binding domain [76]. The homeobox (HB) transcription factors have been classified into 14 classes in plants, which are HD-ZIP I-IV, KNOTTED-1-like homeobox (KNOX), plant homeodomain (PHD), DDT, nodulin homeobox gene (NDX), luminidependens (LD), BEL, wuschel-related homeobox (WOX) and plant interactor homeobox (PINTOX), SAWADEE and plant zinc finger (PLINC) [77].

Recent studies have elucidated that WOX genes play a crucial role in key developmental processes, such as stem cell maintenance and organ development in plants [78,79]. In *A. thaliana*, the WUS protein (*AtWUS*) plays an important role in maintaining the apical meristem of roots and shoots, as well as in the transition from the vegetative to the embryonic phase [80,81]. It also acts as a repressor in stem cell regulation and an activator in floral patterning [82]. Overexpression of *AtWOX1* has been shown to lead to the development of abnormal meristems, which subsequently produce small leaves, low fertility, and dwarfism in *A. thaliana* plants [83,84]. *AtWOX3* plays a key role in lateral organ formation through the recruitment of founder cells [85]. *AtWOX2,* 8, and 9 have been reported to be expressed in early stages of plant development [86]. In rice, *OsWOX11* has been shown to modulate root development, increasing drought resistance, and also participates in the activation of root emergence through the integration of cytokinin–auxin signaling [87,88]. Therefore, these genes are of research interest in plant developmental biology.

Our publication presents studies on the expression of the above-mentioned genes to confirm their involvement in the formation of maize yield. In the case of the LOC100383455 U-box domain-containing protein 7 gene, we observe variable expression levels from 0 to 48 h. It can also be seen that the high-yielding K1 genotype exhibits the greatest fluctuations in this gene’s expression level, especially after 6 h (Figure 10). A similar situation is observed for the LOC103635953 putative WUSCHEL-related homeobox 2 gene. Here, too, in the high-yielding K1 genotype, we observe the greatest fluctuations in this gene’s expression level (Figure 11). Differences in the expression profiles of yield-related genes between cultivars result from a combination of genetic, molecular, and contextual factors. SNPs, INDELs, and promoter duplications can alter transcription factor binding, nucleosome positioning, splicing efficiency, and mRNA stability. Moreover, alleles of genes encoding transcription factors, corepressors, kinases, and hormone receptors modify the regulation of entire gene networks, which influence genes related to yield. The effect of one allele depends on the alleles of other genes (regulatory cascades, protein complexes, metabolic pathways), resulting in nonlinear, context-dependent expression profiles. In different varieties, sequence variants influence DNA methylation and histone modifications, altering gene accessibility. Furthermore, differences in posttranscriptional and posttranslational regulation occur among different genotypes. Consequently, different varieties may pursue different growth and resource allocation strategies (more seeds vs. larger seeds, resistance at the expense of yield, etc.), reflecting their distinct expression profiles.

Our own research and literature reports indicate the usefulness of next-generation sequencing, association mapping, and physical mapping for identifying candidate genes associated with economically important traits in maize. Furthermore, two genes characterized in detail in the publication, LOC100383455 U-box domain-containing protein 7 gene and LOC103635953 putative WUSCHEL-related homeobox 2 gene, may be involved in processes related to maize yield. The literature reports presented above suggest that *PUX7* genes participate in the regulation of growth and development, which indicates their diverse, key role in various biological processes, including yield shaping. The *WOX2* gene modulates signals related to hormones such as gibberellins and cytokinins, which are crucial for seed development, flowering, and grain maturation. This allows for the potential extension of the yield-forming phase or increased grain size through precise control of these pathways.

## 5. Conclusions

Next-generation sequencing (NGS) has revolutionized plant genetics research, offering rapid and relatively inexpensive methods for obtaining sequence data at the genome, transcriptome, and epigenome levels. In the context of the studies presented in this publication, conducted on maize (*Zea mays*), a species of significant economic importance and with a complex genome, NGS enabled the identification of new genes and genetic variants associated with a key agronomic trait, yield. Bioinformatic and functional analyses, such as gene linkage analyses and experimental validation (phenotypic testing under controlled and field conditions), proved essential for translating sequence signals into practical breeding applications. Our own research and literature reports also showed that two selected genes: LOC100383455 U-box domain-containing protein 7 gene and LOC103635953 putative WUSCHEL-related homeobox 2 gene, may be involved in processes related to maize yield. In the case of the LOC100383455 U-box domain-containing protein 7 gene, we observe variable expression levels from 0 to 48 h. It can also be seen that the high-yielding K1 genotype exhibits the greatest fluctuations in this gene’s expression level, especially after 6 h. A similar situation is observed for the LOC103635953 putative WUSCHEL-related homeobox 2 gene. Here, too, in the high-yielding K1 genotype, we observe the greatest fluctuations in this gene’s expression level. Differences in the expression profiles of yield-related genes between cultivars result from a combination of genetic, molecular, and contextual factors. Both genes may be involved in regulating crop height. The *PUX*7 gene is involved in regulating growth and development processes, which may directly impact yield. The *WOX2* gene, on the other hand, functions as a modulator of hormonal signals, particularly those related to gibberellins and cytokines, which play a fundamental role in regulating seed development, flowering, and grain maturation. As a result, the *WOX2* gene may enable extension of the yield-forming phase or increased grain size through precise control of these key signaling pathways. Using NGS to identify new genes associated with economic traits in maize has significant potential to accelerate directional selection, introduce resistance to new stresses, and optimize performance traits through molecular marker selection or direct genetic modification. Key challenges include managing large volumes of data, accurately distinguishing functional from neutral variants, considering environmental and epistatic effects, and addressing the ethical and regulatory aspects of implementing the results in agricultural practice.

## Figures and Tables

**Table 1:** Mean squares from the analysis of variance of the observed traits.

Source of Variation	Hybrids	Residual
Number of degrees of freedom	185	371
Cob length [cm]	2.885 ***	0.4724
Cob diameter [cm]	0.08561 ***	0.01712
Core length [cm]	3.0157 ***	0.4496
Core diameter [cm]	0.059356 ***	0.006924
The number of rows, of grain	7.5731 ***	0.7037
The number of grains in a row	14.708 ***	2.741
Mass of grain from the cob [g]	608.9 ***	230.4
Weight of one thousand grains [g]	4994.3 ***	585.1
Yield from the plot [kg]	1.4167 ***	0.2919
Dry matter content after harvest [t ha^−1^]	7.5033 ***	0.3281
Yield [t ha^−1^]	4.304 ***	1.076

*** *p* < 0.001.

**Table 2:** SilicoDArT and SNP molecular markers significantly associated with the analyzed yield structure traits in Kobierzyce and Smolice (significant associations selected at *p* < 0.001 with correction for multiple testing using the Benjamini–Hochberg method).

Trait	Number of SilicoDArT and SNP Markers	Effect			Percentage of Explained Variation			LOD		
		Min.	Max.	Average	Min.	Max.	Average	Min.	Max.	Average
Cob length [cm]	6564	−0.88	0.905	−0.03	1.7	19.1	8.33	1.34	8.96	4.17
Cob diameter [cm]	2636	−0.153	0.152	−0.01	1.7	16.9	3.87	1.3	7.93	2.26
Core length [cm]	1983	−0.979	1.151	−0.002	1.7	11.8	2.67	1.3	5.65	1.74
Core diameter [cm]	3698	−0.1026	0.1051	0.006	1.7	11.3	2.93	1.3	5.43	1.86
The number of rows of grain	6960	−2.119	2.064	−0.066	1.7	42.1	20.96	1.3	11	8.17
The number of grains in a row	6721	−2.423	2.522	−0.073	1.7	30.9	13.71	1.3	11	6.57
Mass of grain from the cob [g]	1490	−12.98	10.1	−1.35	1.7	16.5	3.41	1.3	7.74	2.06
Weight of one thousand grains [g]	6616	−56.04	56.45	1.27	1.7	47.8	24.54	1.3	11	8.77
Yield from the plot [kg]	1237	−0.659	0.573	−0.015	1.7	17.4	3.66	1.3	8.18	2.17
Dry matter content after harvest	6857	−1.595	1.847	−0.01	1.7	32.7	12.4	1.3	11	5.97
Yield (t ha^−1^)	1114	−1.048	0.976	−0.04	1.7	15.5	3.56	1.3	7.32	2.13
Total	45,876									

**Table 3:** Characteristics and locations of the ten markers most significantly associated (highest LOD values) with all analyzed traits (yield structure trait and yield).

Marker Number	Marker Type	Chromosome Number	Marker Sequence	Location on the Chromosome
459199	Silico DArT	Chr 7, 2701849	TGCAGTATTCTTCCAAAACTGTGAAAAAACTTCACTCCCAAACACCCCCTTAGATGCATGAGATCGGAA	<0.5 Kbp upstream LOC103643803 L-ascorbate oxidase ho olog [*Zea mays*], LOC100282329 uncharacterized <4 Kbp downstream, LOC100282329 [*Zea mays*] (according to Conserved Domains myblike DNA-binding domain, SHAQKYF class) <6K bp upstream LOC100272988 putative TCP-1/cpn60 chaperonin family protein [*Zea mays*] <15 Kbp upstream LOC109939247 putative disease resistance RPP13-like protein 1 [*Zea mays*]
9692004	SNP	Chr 8, 138269978	TGCAGTGACCAGTTTTTCTTTTGGCATTAGAGAACACCCCTTGACATGAGATCGGAAGAGCGGTTCAGC	<In exon 5 of LOC103637325 uncharacterized LOC103637325 [*Zea mays*] <10 Kbp LOC100383831 uncharacterized LOC100383831 [*Zea mays*] <88 Kbp upstream LOC100273381 WAT1-related protein [*Zea mays*]
2447305	Silico DArT	Chr 10, 94117501	TGCAGCTAACCCCAGCCATCCAGAGCGTGGGTGGAGCTGAATTCACTTCACTACCTGCTCTGCATCTGA	<63 Kbp downstream LOC100502418 uncharacterized LOC100502418 [*Zea mays*] <114 Kbp downstream LOC100277156 Tyrosine-specific protein phosphatase-like [*Zea mays*] <204 Kbp upshream LOC100196928 heat shock protein HSP82 [*Zea mays*]
4768759	Silico DArT	Chr 8, 14945867	TGCAGCTCGATCGAAAAAGAGGCTTCTAACATCATCGACCACCAAAACCTCTCGCCCTCTATGGTGGCT	<1.6 Kbp downstream LOC103636090 sphingoid long-chainbase kinase 1 [*Zea mays*] <3.4 Kbp upshream LOC103636089 S-type anion channel SLAH2 [*Zea mays*] <9.4 Kbp downstream, LOC103636092 uncharacterized LOC103636092 [*Zea mays*] <15 Kbp downstream, LOC103636093 probable ADP-ribosylation factor GTPase-activating protein AGD11 [*Zea mays*]
4579916	Silico DArT	Chr 8, 134706585	TGCAGAGGCCCAGGGCTGAAACAGGTAACAGGGGGCCCCCCAGTTTACCCACTGTGCATGAGATCGGAA	<53 Kbp downstream LOC541960 liguleless 4 [*Zea mays*] <68 Kbp upshream LOC100192799 uncharacterized LOC100192799 [*Zea mays*] <123 Kbp downstream LOC100381846 uncharacterized LOC100381846 [*Zea mays*]
4764335	Silico DArT	Chr 10, 95820145	TGCAGGTTGGGGGCAGTTGACCAGGGGAAAGAGATAGAGAGAGGCATGAGATCGGAAGAGCGGTTCAGC	<63 Kbp downstream LOC100502418 uncharacterized LOC100502418 [*Zea mays*] <114 Kbp downstream LOC100277156 Tyrosine specific protein phosphatase-like [*Zea mays*] <204 Kbp upshream LOC100196928 heat shock protein HSP82 [*Zea mays*]
2448946	Silico DArT	Chr 10, 90018580	TGCAGGTTGAGTGCTAGCTTGGGCGTCGTGCCTGGGGTCTGGCGACTTGGATGTTGAGCTGGGCTTCAG	<In exon 4 of LOC100191174 uncharacterized LOC100191174 [*Zea mays*] <1.2 Kbp upstream, LOC100280761 RNA-binding protein 8A [*Zea mays*] <69 Kbp downstream LOC103641365 benzyl alcohol O-benzoyltransferase [*Zea mays*] <87 Kbp downstream LOC103641366 dof zinc finger protein DOF1.6 [*Zea mays*]
2492509	Silico DArT	Chr8, 152554177	TGCAGGACCAAGCTACACCCTTGCCGCAGAATCAGGTATCTACGCTAGGGGTCCAGCATCTGCTAGCAT	<14 Kbp downstream LOC103636139 uncharacterized LOC103636139 <33 Kbp upstream, LOC100281900 uncharacterized LOC100281900 [*Zea mays*] <129 Kbp downstream LOC103636140 carboxyl-terminal-processing peptidase 1, chloroplastic [*Zea mays*] <195 Kbp upstream, LOC100191225 AMSH-like ubiquitin thioesterase 2 [*Zea mays*]
4774802	Silico DArT	Chr8, 135947328	TGCAGCGCTCGCATATATATAATGGATGCAGACATTATACATGAGATCGGAAGAGCGGTTCAGCAGGAA	<300 bp downstream, LOC103635953 putative WUSCHEL-related homeobox 2 [*Zea mays*] <46 Kbp bp downstream LOC100280464 uncharacterized LOC100280464 [*Zea mays*] <62 Kbp bp downstream LOC103637304 uncharacterized LOC103637304 [*Zea mays*] <80 Kbp bp downstream LOC100281170 loricrin [*Zea mays*]
5587791	SNP	Chr8, 170180050	TGCAGAGCCAGCTGGCGGAGGGCGGGAATGGTGGCGGTGCTATTATCGCGTGCCACCACCGGGAGTCGA	<In exon 5 of LOC100383455 U-box domain-containing protein 7 [*Zea mays*] <4 Kbp bp downstream LOC103636398 phospholipase A1 EG1, chloroplastic/mitochondrial [*Zea mays*] <12 Kbp upstream LOC100191367 uncharacterized LOC100191367 [*Zea mays*] <21 Kbp upstream LOC100272376 uncharacterized LOC100272376 [*Zea mays*]

**Table 4:** Sequences of designed primers used to identify newly selected markers significantly associated with the analyzed traits (yield structure feature and yield).

Marker	Marker Type	Polymorphism Identified in the Marker	Primer Names for Polymorphism Identification	Primer Sequences for Polymorphism Identification (5′→3′)	PCR Product (bp)
459199	SilicoDArT—couldn’t design a pair of starters				
9692004	SNP	SNP	9692004_SNP_F	CTCTAATGCCAAAAGAAAAACTGCC	258
			9692004_SNP_R	GTCTGTAAGATCACTATTTAGAGCC	
2447305	SilicoDArT	Insertion + SNP	2447305_DArTiSNP_F	CAGAGCGTGGGTGGAGCCG	969
			2447305_DArTiSNP_R	GTCTGCTTCACTCGAGCCAGAACG	
4768759	SilicoDArT	Deletion	4768759_DArT_F	GATCGGAAGAGCCACCATAG	247
			4768759_DArT_SNP_R	AATAAGCCGATCAAATTCGACGTC	
		SNP	4768759_SNP_F	AGAGGGCGAGAGGTTTGG	229
			4768759_DArT_SNP_R	AATAAGCCGATCAAATTCGACGTC	
4579916	SilicoDArT	SNP	4579916_SNP_F	GCAGAGGCCCAGGGCTGAAACAGTT	407
			4579916_SNP_R	AGCACCAATAAGTACAACACTAAGG	
		Deletion	4579916_SNPDArT_F	CGACAACGAGACCGGCGGCA	221
			4579916_SNPDArT_R	ATGCACAGTGGGTAAACTGGGGGCC	
4764335	SilicoDArT	SNP	4764335_SNP1_F	GACAACATGCCTCTCTCTATCTCGT	130
			4764335_SNP1_R	AATGACAGCTTACCCCTTAATTCTCG	
		SNP	4764335_SNP2_F	ACGTACAGCAGAGTCAACTACCTCT	288
			4764335_SNP2_R	CAGGTTGGGGGCAGTTGACCGG	
2448946	SilicoDArT	SNP	2448946_SNP_F	GGTTGAGTGCTAGCTTGGCC	280
			2448946_SNP_R	ATCTTCACTGACCTATCTCAAAAC	
2492509	SilicoDArT	SNP + Deletion	2492509_SNPiDel1_F	CTGGTCGCGTGCCTCGTCACC	217
			2492509_SNPiDel1_R	TTGCCGCAGAATCAGGTATCTACAC	
		Deletion	2492509_Del2_F	TGCGGCAAGGGTGTAGCTTGG	360
			2492509_Del2_R	AGATAGAAATAAACCCCACTCCATTGG	
4774802	SilicoDArT	SNP + Deletion	4774802_SNPDel_F	TGCAGTACACATGTCCTTC	121
			4774802_SNPDel_R	CGCATATATATAATGGATGAA	
		Insertion	4774802_Ins_F	CCATTATATATATGCGAGCG	225
			4774802_Ins_R	TCAGTTTGTTTGGTTGTAAGTTG	
5587791	SNP	SNP	5587791_SNP_F	TATCGCGTGCCACCACCGGGAGTTC	282
			5587791_SNP_R	CCGAGGAGGTGGGGGAAGAAC	

## Data Availability

The data presented in this study are available on request from the corresponding author.

## Supplementary Materials

The following supporting information can be downloaded at: https://www.mdpi.com/article/10.3390/cimb47121008/s1.

## References

[1] Erenstein O., Chamberlin J., Sonder K. (2021). Estimating the global number and distribution of maize and wheat farms. Glob. Food Secur..

[2] Erenstein O., Jaleta M., Sonder K., Mottaleb K., Prasanna B.M. (2022). Global maize production, consumption and trade: Trends and R&D implications. Glob. Food Secur..

[3] Martinez-Feria R.A., Basso B. (2020). Unstable crop yields reveal opportunities for site-specific adaptations to climate variability. Sci. Rep..

[4] Long Y., Wang C., Liu C., Li H., Pu A., Dong Z., Wei X., Wan X. (2024). Molecular mechanisms controlling grain size and weight and their biotechnological breeding applications in maize and other cereal crops. J. Adv. Res..

[5] Kumar V., Chand K., Suman M., Pandey H.C. (2017). Economic analysis of Maize Seed Production on Farmers’ Fields. Agro-Economist.

[6] Guo W., Lian T., Wang B., Guan J., Yuan D., Wang H., Azam F.M.S., Wan X., Wang W., Liang Q. (2019). Genetic mapping of folate QTLs using a segregated population in maize (*Zea mays* L.). J. Integr. Plant Biol..

[7] Zhang H., Lu Y., Ma Y., Fu J., Wang J. (2021). Genetic and molecular control of grain yield in maize. Mol. Breed..

[8] Zhu M., Tong L., Xu M., Zhong T. (2021). Genetic dissection of maize disease resistance and its applications in molecular breeding. Mol. Breed..

[9] Xu Y., Li P., Yang Z., Xu C. (2017). Genetic mapping of quantitative trait loci in crops. Crop J..

[10] Zenke-Philippi C., Frisch M., Thiemann A., Seifert F., Schrag T., Melchinger A.E., Herzog E. (2017). Transcriptome-based prediction of hybrid performance with unbalanced data from a maize breeding programme. Plant Breed..

[11] Cooper M., Technow F., Messina C., Gho C., Totir L.R. (2016). Use of crop growth models with whole-genome prediction: Application to a maize multienvironment trial. Crop Sci..

[12] Metzker M.L. (2010). Sequencing technologies—The next generation. Nat. Rev. Genet..

[13] Poplin R., Chang P.C., Alexander D., Schwartz S., Colthurst T., Ku A., Newburger D., Dijamco J., Nguyen N., Afshar P.T. (2018). A universal SNP and small indel variant caller using deep neural networks. Nat. Biotechnol..

[14] Buenrostro J.D., Giresi P.G., Zaba L.C., Chang H.Y., Greenleaf W.J. (2013). Transposition of na-tive chromatin for fast and sensitive epigenomic profiling of open chromatin. Nat. Methods.

[15] Wenger A.M., Peluso P., Rowell W.J., Chang P.C., Hall R.J., Concepcion G.T., Ebler J., Fungtammasan A., Kolesnikov A., Olson N.D. (2019). Accurate circular consensus long-read sequencing improves variant detection and assembly of a human genome. Nat. Biotechnol..

[16] Jain M., Koren S., Miga K.H., Quick J., Rand A.C., Sasani T.A., Tyson J.R., Beggs A.D., Dilthey A.T., Fiddes I.T. (2018). Nanopore sequencing and assembly of a human genome with ultra-long reads. Nat. Biotechnol..

[17] Barilli E., Cobos M.J., Carrillo E., Kilian A., Carling J., Rubiales D. (2018). A High-Density Integrated DArTseq SNP-Based Genetic Map of Pisum fulvum and Identification of QTLs Controlling Rust Resistance. Front. Plant Sci..

[18] Alam M., Neal J., O’Connor K., Kilian A., Topp B. (2018). Ultra-high-throughput DArTseq-based silicoDArT and SNP markers for genomic studies in macadamia. PLoS ONE.

[19] Courtois B., Audebert A., Dardou A., Roques S., Ghneim-Herrera T., Droc G., Frouin J., Rouan L., Gozé E., Kilian A. (2013). Genome-Wide Association Mapping of Root Traits in a Japonica Rice Panel. PLoS ONE.

[20] Cruz V.M.V., Kilian A., Dierig D.A. (2013). Development of DArT Marker Platforms and Genetic Diversity Assessment of the U.S. Collection of the New Oilseed Crop Lesquerella and Related Species. PLoS ONE.

[21] Sakhare A.S., Kota S., Rathod S., Parmar B., Chinnusamy V. (2022). Genome-Wide Association Study. Genotyping by Sequencing for Crop Improvement.

[22] Abdurakhmonov I.Y., Abdukarimov A. (2008). Application of Association Mapping to Understanding the Genetic Diversity of Plant Germplasm Resources. Int. J. Plant Genom..

[23] Rafalski J.A. (2010). Association genetics in crop improvement. Curr. Opin. Plant Biol..

[24] Zhu C., Gore M., Buckler E.S., Yu J. (2008). Status and Prospects of Association Mapping in Plants. Plant Genome.

[25] Rakoczy-Trojanowska M., Krajewski P., Bocianowski J., Schollenberger M., Wakuliński W., Milczarski P., Masojć P., Targońska-Karasek M., Banaszak Z., Banaszak K. (2017). Identification of Single Nucleotide Polymorphisms Associated with Brown Rust Resistance, α-Amylase Activity and Pre-harvest Sprouting in Rye (*Secale cereale* L.). Plant Mol. Biol. Rep..

[26] Bar-Hen A., Charcosset A., Bourgoin M., Guiard J. (1995). Relationship between genetic markers and morphological traits in a maize inbred lines collection. Euphytica.

[27] Pritchard J.K. (2001). Deconstructing Maize Population Structure. Nat. Genet..

[28] Dhliwayo T., Pixley K., Menkir A., Warburton M. (2009). Combining Ability, Genetic Distances, and Heterosis among Elite CIMMYT and IITA Tropical Maize Inbred Lines. Crop Sci..

[29] Chander S., Guo Y.Q., Yang X.H., Zhang Z., Lu X.Q., Yan J.B., Song T.M., Rocheford T.R., Li J.S. (2008). Using molecular markers to identify two major loci controlling carotenoid contents in maize grain. Theor. Appl. Genet..

[30] Messing J., Bharti A.K., Karlowski W.M., Gundlach H., Kim H.R., Yu Y., Wei F., Fuks G., Soderlund C.A., Mayer K.F.X. (2004). Sequence composition and genome organization of maize. Proc. Natl. Acad. Sci. USA.

[31] Baird N.A., Etter P.D., Atwood T.S., Curey M.C., Shiver A.L., Lewis Z.A., Selker E.U., Cres-ko W.A., Johnson E.A. (2008). Rapid SNP Discovery and Genetic Mapping Using Sequenced RAD Markers. PLoS ONE.

[32] Guo Z., Tucker D.M., Wang D., Basten C.J., Ersoz E., Briggs W.H., Lu J., Li M., Gay G. (2013). Accuracy of Across-Environment Genome-Wide Prediction in Maize Nested Association Mapping Populations. Genes Genomes Genet..

[33] Benke A., Urbany C., Stich B. (2015). Genome-wide association mapping of iron homeostasis in the maize association population. BMC Genet..

[34] Prasanna B.M., Pixley K., Warburton M., Xie C.-X. (2010). Molecular marker-assisted breeding options for maize improvement in Asia. Mol. Breed..

[35] Beavis W.D., Smith O.S., Grant D., Fincher R.R. (1994). Identyfication of quantitative trait loci using a small sample of topcrossed and F4 progeny from maize. Crop Sci..

[36] Ribaut J., Hoisington D.A., Deutsch J.A., Jiang C., Gonzalez-de-Leon D. (1996). Identyfication of quantitative trait loci under drought conditions in tropical maize. Flowering parameters and the anthesis silking interval. Theor. Appl. Genet..

[37] Veldboom L., Lee M. (1994). Molecular-marker-facicitated studies of morphological traits in maize. De-termination of QTLs for grain yield components. Theor. Appl. Genet..

[38] Austin D.F., Lee M., Veldboom L.R. (2001). Genetic mapping in maize with hybrid progeny across testers and generations: Grain yield and grain moisture. Crop Sci..

[39] Melchinger A.E., Utz H., Schön C.C. (1998). Quantitative trait locus (QTL) mapping using different testers and independent population samples in maize reveals low power of QTL detection and large bias in estimates of QTL effects. Genetics.

[40] Tang J., Yan Y., Ma X., Teng W., Wu W., Dai J., Dhillon B.S., Melchinger A.E., Li J. (2009). Dissection of the genetic basis of heterosis in an elite maize hybrid by QTL mapping in an immortalized F2 population. Theor. Appl. Genet..

[41] Yang S., Pang W., Ash G., Harper J., Carling J., Wenzl P., Huttner E., Zong X., Kilian A. (2006). Low level of genetic diversity in cultivated Pigeonpea compared to its wild relatives is revealed by diversity arrays technology. Theor. Appl. Genet..

[42] Wenzl P., Carling J., Kudrn D., Jaccoud D., Huttner E., Kleinhofs A., Kilian A. (2004). Diversity Arrays Technology (DArT) for whole-genome profiling of barley. Proc. Natl. Acad. Sci. USA.

[43] Bocianowski J., Leśniewska-Bocianowska A. (2025). Towards the Identification of Candidate Genes for Pollen Morphological Traits in Rubus L. Using Association Mapping. Forests.

[44] van Eeuwijk F.A., Bink M.C.A.M., Chenu K., Chapman S.C. (2010). Detection and use of QTL for complex traits in multiple environments. Curr. Opin. Plant Biol..

[45] Malosetti M., Ribaut J.-M., van Eeuwijk F.A. (2013). The statistical analysis of multi-environment data: Modeling genotype-by-environment interaction and its genetic basis. Front. Physiol..

[46] VSN International (2023). VSN International Genstat for Windows.

[47] Patterson N., Price A.L., Reich D. (2006). Population structure and eigenanalysis. PLoS Genet..

[48] Wan W., Kim S., Castel B., Charoennit N., Chae E. (2021). Genetics of Autoimmunity in Plants: An Evolutionary Genetics Perspective. New Phytol..

[49] Liu H., Niu Y., Gonzalez-Portilla P.J., Zhou H., Wang L., Zuo T., Qin C., Tai S., Jansen C., Shen Y. (2015). An Ultra-HighDensity Map as a Community Resource for Discerning the Genetic Basis of Quantitative Traits in Maize. BMC Genom..

[50] Andorf C.M., Cannon E.K., Portwood J.L., Gardiner J.M., Harper L.C., Schaeffer M.L., Braun B.L., Campbell D.A., Vinnakota A.G., Sribalusu V.V. (2016). Maize GDB Update: New Tools, Data and Interface for the Maize Model Organism Database. Nucleic Acids Res..

[51] Araus J.L., Cairns J.E. (2014). Field high-throughput phenotyping: The new crop breeding frontier. Trends Plant Sci..

[52] Godfray H.C.J., Goldin I. (2014). How can 9–10 billion people be fed sustainably and equitably by 2050. Is the Planet Full.

[53] Shendure J., Ji H. (2008). Next-generation DNA sequencing. Nat. Biotechnol..

[54] Michael T.P., VanBuren R. (2015). Progress, challenges and the future of crop genomes. Curr. Opin. Plant Biol..

[55] Bocianowski J., Tomkowiak A., Bocianowska M., Sobiech A. (2023). The use of DArTseq technology to identify markers related to the heterosis effects in selected traits in maize. Curr. Issues Mol. Biol..

[56] Sobiech A., Tomkowiak A., Nowak B., Bocianowski J., Wolko Ł., Spychała J. (2022). Associative and Physical Mapping of Markers Related to Fusarium in Maize Resistance, Obtained by Next-Generation Sequencing (NGS). Int. J. Mol. Sci..

[57] Soto J., Rodriguez-Antolin C., Vallespín E., de Castro Carpeño J., Ibanez de Caceres I. (2016). The Impact of Next-Generation Sequencing on the DNA Methylation–Based Translational Cancer Research. Transl. Res..

[58] Addo-Quaye C., Buescher E., Best N., Chaikam V., Baxter I., Dilkes B.P. (2017). Forward Genetics by Sequencing EMS Variation Induced Inbred Lines. G3 Genes Genomes Genet..

[59] Anandhakumar C., Kizaki S., Bando T., Pandian G.N., Sugiyama H. (2015). Advancing Small-Molecule-Based Chemical Biology with Next-Generation Sequencing Technologies. ChemBioChem.

[60] Michael C.R. (2009). Alavanja Introduction: Pesticides Use and Exposure, Extensive Worldwide. Rev. Environ. Health.

[61] Perez-de-Castro A.M., Vilanova S., Canizares J., Pascual L., Blanca J.M., Diez M.J., Prohens J., Pico B. (2012). Application of Genomic Tools in Plant Breeding. Curr. Genom..

[62] Huang X., Zhao Y., Wei X., Li C., Wang A., Zhao Q., Li W., Guo Y., Deng L., Zhu C. (2011). Ge-nome-Wide Association Study of Flowering Time and Grain Yield Traits in a Worldwide Collection of Rice Germplasm. Nat. Genet..

[63] Ding J., Ali F., Chen G., Li H., Mahuku G., Yang N., Narro L., Magorokosho C., Makumbi D., Yan J. (2015). Genome-Wide Association Mapping Reveals Novel Sources of Resistance to Northern Corn Leaf Blight in Maize. BMC Plant Biol..

[64] Jiao Y., Peluso P., Shi J., Liang T., Stitzer M.C., Wang B., Campbell M.S., Stein J.C., Wei X., Chin C. (2017). Improved Maize Reference Genome with Single-Molecule Technologies. Nature.

[65] Du Z., Zhou X., Li L., Su Z. (2009). A database of plants’ ubiquitin proteasome system. BMC Genom..

[66] Wang D.R., Zhang X.W., Xu R.R., Wang G.L., You C.X., An J.P. (2022). Apple U-box-type E3 ubiquitin ligase MdPUB23 reduces cold-stress tolerance by degrading the cold-stress regulatory protein MdICE1. Hortic. Res..

[67] Hu H., Dong C., Sun D., Hu Y., Xie J. (2018). Genome-wide identification and analysis of U-box E3 ubiquitin-protein ligase gene family in banana. Int. J. Mol. Sci..

[68] Tan B., Lian X., Cheng J., Zeng W., Zheng X., Wang W., Ye X., Li J., Li Z., Zhang L. (2019). Genome-wide identification and transcriptome profiling reveal that E3 ubiquitin ligase genes relevant to ethylene, auxin, and abscisic acid are differentially expressed in the fruits of melting flesh and stony hard peach varieties. BMC Genom..

[69] Sharma B., Taganna J. (2020). Genome-wide analysis of the U-box E3 ubiquitin ligase enzyme gene family in tomato. Sci. Rep..

[70] Kim M.S., Kang K.K., Cho Y.G. (2021). Molecular and functional analysis of U-box E3 ubiquitin ligase gene family in rice (Oryza sativa). Int. J. Mol. Sci..

[71] Zeng L.R., Park C.H., Venu R.C., Gough J., Wang G.L. (2008). Classification, expression pattern, and E3 ligase activity assay of rice U-box-containing proteins. Mol. Plant.

[72] Trujillo M. (2018). News from the PUB: Plant U-box type E3 ubiquitin ligases. J. Exp. Bot..

[73] Chen X., Wang T., Rehman A.U., Wang Y., Qi J., Li Z., Song C., Wang B., Yang S., Gong Z. (2021). Arabidopsis U-box E3 ubiquitin ligase PUB11 negatively regulates drought tolerance by degrading the receptor-like protein kinases LRR1 and KIN7. J. Integr. Plant Biol..

[74] Trenner J., Monaghan J., Saeed B., Quint M., Shabek N., Trujillo M. (2020). Evolution and functions of plant U-box proteins: From protein quality control to signaling. Ann. Rev. Plant Biol..

[75] Li J., Zhang Y., Gao Z., Xu X., Wang Y., Lin Y., Ye P., Huang T. (2021). Plant U-box E3 ligases PUB25 and PUB26 control organ growth in arabidopsis. New Phytol..

[76] Alvarez J.M., Bueno N., Cañas R.A., Avila C., Cánovas F.M., Ordás R.J. (2018). Analysis of the WUSCHEL-RELATED HOMEOBOX gene family in Pinus pinaster: New insights into the gene family evolution. Plant Physiol. Biochem..

[77] Mukherjee K., Brocchieri L., Bürglin T.R. (2009). A comprehensive classification and evolutionary analy-sis of plant homeobox genes. Mol. Biol. Evol..

[78] Schoof H., Lenhard M., Haecker A., Mayer K.F., Jürgens G., Laux T. (2000). The stem cell population of arabidopsis shoot meristems is maintained by a regulatory loop between the CLAVATA and WUSCHEL genes. Cell.

[79] Ueda M., Zhang Z., Laux T. (2011). Transcriptional activation of arabidopsis axis patterning genes WOX8/9 links zygote polarity to embryo development. Dev. Cell.

[80] Mayer K.F., Schoof H., Haecker A., Lenhard M., Jürgens G., Laux T. (1995). Role of WUSCHEL in regulating stem cell fate in the arabidopsis shoot meristem. Cell.

[81] He P., Zhang Y., Liu H., Yuan Y., Wang C., Yu J., Xiao G. (2019). Comprehensive analysis of WOX genes uncovers that WOX13 is involved in phytohormone-mediated fiber development in cotton. BMC Plant Biol..

[82] Ikeda M., Mitsuda N., Ohme-Takagi M. (2009). Arabidopsis WUSCHEL is a bifunctional transcription factor that acts as a repressor in stem cell regulation and as an activator in floral patterning. Plant Cell.

[83] Graaff E.V.D., Laux T., Rensing S.A. (2009). The WUS homeobox-containing (WOX) protein family. Genome Biol..

[84] Zhang Y., Wu R., Qin G., Chen Z., Gu H., Qu L.J. (2011). Over-expression of WOX1 leads to defects in meristem development and polyamine homeostasis in arabidopsis^F^. Integr. Plant Biol..

[85] Shimizu R., Ji J., Kelsey E., Ohtsu K., Schnable P.S., Scanlon M.J. (2009). Tissue specificity and evolution of meristematic WOX3 function. Plant Physiol..

[86] Wang P., Guo Y., Chen X., Zheng Y., Sun Y., Yang J., Ye N. (2019). Genome-wide identification of WOX genes and their expression patterns under different hormone and abiotic stress treatments in tea plant (Camellia sinensis). Trees.

[87] Zhao Y., Hu Y., Dai M., Huang L., Zhou D.X. (2009). The WUSCHEL-related homeobox gene WOX11 is required to activate shoot-borne crown root development in rice. Plant Cell.

[88] Cheng S., Zhou D.X., Zhao Y. (2016). WUSCHEL-related homeobox gene WOX11 increases rice drought resistance by controlling root hair formation and root system development. Plant Signal Behav..

