# Supplementary material for: Using Genome-Wide Association Studies to Reveal DArTseq and SNP Loci Associated with Agronomic Traits and Yield in Maize
Source: Curr Issues Mol Biol. 2025 Nov 30;47(12):1008. doi: 10.3390/cimb47121008 (PMC12731560; doi:10.3390/cimb47121008)
Supplement: Supplementary file 1 [file cimb-47-01008-s001.zip › cimb-3968602-supplementary.pdf]

Table S1. Average values of the observed traits for individual hybrids.

Hybrid	Cob length [cm]		Cob diameter [cm]		Core length [cm]		Core diameter [cm]		The number of rows of grain		The number of grains in a row		Mass of grain from the cob [g]	
	Mean	s.e.	Mean	s.e.	Mean	s.e.	Mean	s.e.	Mean	s.e.	Mean	s.e.	Mean	s.e.
G15.18	17.17	0.1667	4.378	0.14699	16.11	0.294	2.311	0.05556	16.44	0.5879	33.01	0.6723	137.5	13.575
G15.19	17.28	0.0556	4.6	0.05774	15.89	0.2778	2.456	0.0294	14.22	0.5879	32.89	0.4843	169.4	3.212
G15.20	18.5	0.0962	4.6	0.03849	17.33	0.1667	2.378	0.08012	15.56	0.5879	36.11	0.4006	190.8	2.069
G15.21	16.61	0.6111	4.2	0.13333	15.44	0.4843	2.244	0.05879	15.11	0.4444	31.67	1.3333	134.4	15.552
G16.01	17.83	0.3849	4.322	0.06186	16.56	0.0556	2.256	0.02222	17.78	0.4444	34.67	0.5092	150.7	10.393
G16.02	17.22	1.1153	4.667	0.10184	16.33	1.2509	2.511	0.01111	15.56	0.2222	30.67	2.5459	160.3	20.842
G16.03	18.5	1.3642	4.511	0.09493	17.44	1.4917	2.489	0.06186	17.78	0.2222	34.78	2.9144	167.8	19.323
G16.04	16.83	0.1667	4.556	0.06186	16.06	0.0556	2.578	0.05556	14.44	0.4444	30.44	0.7286	152.1	7.762
G16.05	18.89	0.4938	4.467	0.01925	18.17	0.5774	2.533	0.03849	17.11	0.4444	33.67	1.3878	163.4	10.665
G16.06	17.22	0.2003	4.567	0.05092	16.33	0.1667	2.522	0.02222	14.89	0.5879	32.33	0.6939	159.7	2.119
G16.07	18.06	0.1111	4.456	0.07778	17.33	0	2.389	0.06186	16.22	0.2222	36.89	0.2222	163	8.738
G16.08	18.83	0.3333	4.556	0.04006	17.89	0.2222	2.533	0.01925	15.11	0.8012	35.22	0.9876	173	1.03
G16.09	20.11	0.53	4.422	0.04006	19.17	0.5092	2.544	0.01111	16.67	0.6667	38.33	0.8819	173.1	4.197
G16.10	16.5	0.5092	4.578	0.04843	16.06	0.4006	2.589	0.05556	15.11	0.2222	31.56	0.8678	153.4	8.522
G16.11	18.17	0.1925	4.678	0.06186	17.28	0.2003	2.6	0.01925	17.56	0.2222	35.56	0.9095	176.8	2.824
G16.12	17.39	0.3889	4.756	0.08889	16.33	0.3469	2.656	0.04006	15.56	0.2222	32.11	0.7286	173.7	9.027
G16.13	17.89	0.4843	4.667	0.03849	17.22	0.5472	2.644	0.01111	17.11	0.5879	36.78	0.6759	162.1	9.183
G16.14	17.33	0.1667	4.878	0.02222	16.28	0.2003	2.589	0.0294	14.67	0	35.56	0.9095	183.9	6.215
G16.15	17.39	0.3643	4.844	0.06759	16.33	0.3469	2.444	0.05879	17.78	0.5879	38.78	1.9468	188	8.367
G16.16	18.39	0.2778	4.611	0.0294	17.89	0.0556	2.578	0.04843	15.78	0.5879	32.78	0.4006	169.4	2.016
G16.17	20.11	0.2422	4.4	0.01925	19.33	0.2546	2.489	0.0294	17.33	0.3849	35.78	0.5556	177.5	5.332
G16.18	16.94	0.2422	4.533	0.05774	16.22	0.2422	2.5	0.03333	13.78	0.4444	32.22	0.4444	155.3	5.943
G16.19	18.28	0.4938	4.644	0.05879	17.72	0.4843	2.544	0.01111	16.89	0.8012	36	0.6939	170.5	4.049
G16.20	15.39	0.655	4.678	0.05879	14.62	0.4656	2.511	0.02222	15.33	0.6667	29.44	1.1277	155.5	9.793
G16.21	17.28	0.3889	4.8	0.1	16.56	0.2422	2.611	0.06186	17.33	0.3849	34.33	0.3849	175.4	9.29
G17.01	17.67	0.2546	4.633	0.05774	17.11	0.3889	2.778	0.04843	13.56	0.5879	35.11	0.5879	166.2	17.904
G17.02	17.78	0.2222	4.622	0.07778	16.89	0.3889	2.656	0.04006	15.33	0.3849	36.22	0.5879	166.3	8.834
G17.04	18.89	0.5879	4.633	0.09623	17.78	0.7718	2.656	0.02222	16	0.7698	34.44	1.4186	182.8	2.786
G17.05	17.83	0.3469	4.744	0.04006	17	0.2887	2.5	0.03849	13.78	0.2222	33.67	0.8819	179.2	8.018
G17.06	19	0.5092	4.433	0.05092	18.06	0.4747	2.4	0.03333	14.67	0.3849	36.78	0.7286	172.4	0.842
G17.07	16.11	0.1111	4.667	0.05092	15.06	0.2003	2.522	0.0294	14.89	0.2222	31.67	0.3849	161	5.994
G17.08	17.06	0.3889	4.678	0.02222	16.06	0.3379	2.511	0.0294	16.89	0.2222	34.22	0.5556	164.1	3.571
G17.09	16.33	0.0962	4.578	0.04006	15.28	0.147	2.7	0.03333	16.44	0.2222	28.22	0.6186	152.6	1.318
G17.10	17.22	0.147	4.711	0.07778	16.11	0.147	2.778	0.01111	18.89	0.4444	33.11	0.7778	166.5	3.623
G17.11	17.06	0.2222	4.833	0.09623	15.78	0.2003	2.567	0.01925	17.78	0.5879	33	0.7698	161.3	10.943
G17.12	17	0.1925	4.789	0.04843	15.89	0.0556	2.456	0.04843	18.67	0.3849	37.11	0.294	162.4	3.782
G17.13	17.72	0.0556	4.989	0.10599	16.89	0.0556	2.689	0.04006	16.67	0.3849	35.56	0.4444	184.6	12.026
G17.14	17.17	0.441	4.778	0.04843	16.17	0.4811	2.611	0.04444	18.67	0.3849	36	1.3878	154.3	9.932
G17.15	17.44	0.2422	4.544	0.08678	16.56	0.4006	2.433	0.03849	14.22	0.5879	33.44	0.6759	162.4	3.642
G17.16	18.06	0.2422	4.644	0.07778	16.78	0.2422	2.433	0.03849	17.56	0.2222	35.22	0.4006	170.4	6.336
G17.17	16.89	0.4843	4.622	0.06186	15.83	0.4811	2.567	0.03849	14.89	0.2222	34.78	0.9095	158.8	8.245
G17.18	17.28	0.3093	4.744	0.10943	16.17	0.2546	2.567	0.01925	17.22	0.6186	38.89	0.4843	170.3	11.528
G17.19	16.83	0.2546	5.089	0.07778	15.94	0.2222	2.967	0.05774	15.78	0.4444	32.33	0.5092	186	8.391
G17.20	16.89	0.4843	4.933	0	15.83	0.4811	2.889	0.04006	17.78	0.5879	33.78	1.0599	169.9	2.425
G17.21	17.94	0.53	4.756	0.07286	17.11	0.6186	2.6	0.03849	15.11	0.5879	33.33	1.0715	186.6	11.028
G18.01	17.67	0.1925	4.611	0.05879	17.11	0.3379	2.544	0.04444	17.33	0.3849	34.89	0.7286	162.7	2.82

G18.02	15.94	0.0556	4.511	0.01111	15.17	0.1667	2.344	0.04006	13.33	0	32.78	0.4843	155.7	1.779
G18.03	17.39	0.7222	4.611	0.14948	16.28	0.53	2.489	0.04444	17.11	0.2222	33.67	1.3472	159.2	20.2
G18.04	14.89	0.3093	4.633	0	14.17	0.2546	2.567	0	14.89	0.4444	29.22	0.4444	139.6	3.745
G18.05	16.67	0.5358	4.744	0.0294	16.11	0.2422	2.611	0.08012	17.33	0	33.78	1.16	169.3	7.443
G18.06	16.44	0.3093	4.5	0.06939	15.78	0.2422	2.544	0.04006	13.33	0.3849	29.44	0.7286	149	11.15
G18.07	17.39	0.147	4.456	0.11277	16.28	0.147	2.378	0.10599	14.89	0.5879	31.56	1.0599	151.8	12.274
G18.08	17.67	0.1667	4.611	0.04843	17.06	0.2222	2.611	0.01111	16.44	0.4444	35.44	1.1111	173.5	2.516
G18.09	18.39	0.7778	4.811	0.06186	17.61	0.8184	2.711	0.04444	20.22	0.5879	38	1.3472	185.7	9.239
G18.10	16.18	0.2655	4.889	0.10599	14.83	0.2546	2.711	0.04843	17.11	0.5879	30.78	0.8012	173	5.074
G18.11	17.83	0.0962	4.789	0.02222	16.72	0.0556	2.611	0.01111	19.11	0.5879	35.44	0.4006	180.5	6.672
G18.12	16.89	0.4747	4.478	0.02222	16.17	0.6009	2.467	0.06939	13.33	0	31.22	0.4006	158.5	2.222
G18.13	17.17	0.2546	4.722	0.14444	16.11	0.2778	2.644	0.07286	16.67	1.0184	32.22	0.294	168.1	9.528
G18.14	16.72	0.4339	4.556	0.11759	16.06	0.5879	2.467	0.03849	13.11	0.5879	33.44	0.9095	161.9	2.047
G18.15	17.89	0.0556	4.511	0.05879	17	0.1667	2.4	0.03333	15.78	0.2222	37	0.3849	173.6	3.068
G18.16	17.17	0.441	4.456	0.04843	16.39	0.3889	2.467	0.05092	13.78	0.2222	34.56	0.5879	152.4	8.584
G18.17	18.5	0.1667	4.456	0.0294	17.72	0.0556	2.611	0.04444	17.78	0.2222	37.11	0.294	158.6	5.39
G18.18	16.67	0.1667	4.544	0.04006	14.83	0.8333	2.5	0.01925	14	0.7698	31.33	0.3333	164.4	4.487
G18.19	17.4	0.4482	4.444	0.04444	16.33	0.4811	2.378	0.01111	15.56	0.5879	35.56	0.8678	161.5	8.476
G18.20	17	0.2887	4.622	0.0294	16.17	0.3333	2.644	0.07778	13.11	0.2222	30.56	1.2814	147.3	8.433
G18.21	18.61	1.1399	4.633	0.1262	17.78	1.0015	2.633	0.05092	14.89	0.5879	33.67	1.3472	168.9	18.857
G19.01	17.67	0.1667	4.722	0.0294	16.78	0.1111	2.622	0.02222	15.78	0.2222	33.11	0.4006	185.8	7.841
G19.02	18.56	0.2778	4.622	0.01111	17.61	0.4747	2.456	0.02222	17.56	0.2222	36.78	0.1111	183.1	7.743
G19.03	17.22	0.294	4.411	0.12373	16.56	0.4339	2.5	0.03333	15.11	0.5879	32.89	0.5879	158.8	10.035
G19.04	18.17	0	4.5	0.10715	17.39	0.0556	2.467	0.05092	16.67	0.7698	34.67	0.1925	174.2	10.956
G19.05	15.94	0.3643	4.478	0.16368	15.28	0.3643	2.544	0.07778	13.78	0.8012	31.56	0.4444	145.7	12.729
G19.06	17.56	0.0556	4.522	0.07286	16.44	0.147	2.367	0.05774	15.33	0.3849	35.89	0.4843	166.9	10.361
G19.07	20	0.3469	4.6	0.03333	19.06	0.2778	2.544	0.02222	15.78	0.2222	35	0.6939	196.1	8.554
G19.08	18.72	0.2222	4.389	0.06759	17.94	0.3379	2.4	0.05092	16.44	0.5879	35.22	0.9686	171.2	16.634
G19.09	16.94	0.2003	4.667	0.06939	15.89	0.147	2.456	0.02222	14.22	0.8012	31.11	0.1111	176.3	5.391
G19.10	18.06	0.147	4.744	0.04006	17.28	0.1111	2.544	0.0294	16.89	0.2222	35.11	0.1111	192.8	12.336
G19.11	16.67	0.1925	4.578	0.0294	15.72	0.1111	2.6	0.05092	15.11	0.2222	29.78	0.7778	169.1	11.592
G19.12	17.28	0.147	4.7	0.05092	16.5	0.0962	2.633	0.06939	16	0.3849	33.44	0.4843	190.5	6.735
G19.13	17.22	0.294	4.633	0.06667	16.33	0.3333	2.5	0	13.78	0.5879	32.89	0.2222	179.6	10.819
G19.14	17.22	0.3643	4.611	0.06186	16.33	0.2546	2.433	0.03333	15.11	0.2222	34.89	1.1277	177.2	17.225
G19.15	18.28	0.5556	4.444	0.16368	17.33	0.5	2.578	0.08012	14	0.7698	33	0.3849	162.3	1.785
G19.16	19.44	0.2422	4.344	0.07286	18.44	0.2422	2.522	0.09493	16.22	0.8012	35.78	0.9095	174.9	1.571
G19.17	18.22	0.2422	4.689	0.04006	17.61	0.0556	2.6	0.05092	14.22	0.2222	34.89	1.0599	189.5	15.138
G19.18	18.72	0.0556	4.611	0.01111	18	0.0962	2.6	0.03333	16	0.3849	35.33	0.5092	186.1	12.061
G19.19	18.11	0.3093	4.622	0.10943	17.56	0.2003	2.489	0.02222	13.78	0.2222	35	0.3849	189.8	4.634
G19.20	18.28	0.147	4.567	0.03333	17.5	0.1667	2.422	0.04843	15.33	0.3849	35.89	0.7778	186.8	3.827
G19.21	17.33	0.0962	4.578	0.02222	16.67	0.0962	2.467	0.06939	15.78	0.2222	34.33	1.1706	164.4	3.591
G20.01	19	0.0962	4.478	0.0294	18.17	0.1667	2.544	0.04444	18	0.3849	38.11	0.2222	177.1	8.903
G20.02	16.5	0.8221	4.5	0.03333	17.28	0.147	2.456	0.04006	15.56	0.2222	37.11	0.7286	179.4	3.316
G20.03	18.39	0.4843	4.422	0.06759	17.78	0.147	2.633	0.01925	18.89	0.4444	38.11	0.6759	164.2	6.237
G20.05	17.94	0.147	4.589	0.06186	17.33	0.0962	2.622	0.02222	17.33	0.3849	33.11	0.9493	165	3.985
G20.06	17.61	0.0556	4.767	0.06667	17	0.1667	2.667	0.01925	15.78	0.8889	33.22	0.9686	180.7	2.829
G20.07	18	0.3333	4.556	0.05879	17.61	0.4843	2.656	0.06759	17.11	0.2222	35	0.6939	172.6	4.514
G20.09	15.94	0.2422	4.611	0.04006	15.61	0.3093	2.489	0.0294	16.44	0.5879	31.44	1.2373	149.4	5.219
G20.10	17.44	1.0289	4.444	0.05879	16.33	0.5092	2.511	0.05879	14.22	0.4444	32.11	1.0599	157.8	9.253
G20.11	19.28	0.8012	4.544	0.05879	18.39	0.53	2.556	0.04006	16.67	0.3849	35.89	0.8678	183.3	6.849
G20.12	16.39	0.3093	4.456	0.01111	16	0.3333	2.544	0.01111	14.89	0.2222	34.89	0.4006	151.6	7.747

G20.13	17.28	0.147	4.433	0.01925	16.56	0.0556	2.567	0.05092	16.22	0.5879	37.56	0.2222	160.7	1.946
G20.14	16	0.3849	4.211	0.04444	15.33	0.4194	2.344	0.04843	13.56	0.4444	32.78	0.7778	136.6	9.777
G20.15	17.56	0.2003	4.156	0.01111	16.83	0.2546	2.278	0.04006	15.78	0.2222	31.18	4.0818	146	6.016
G20.16	16.39	0.4747	4.611	0.06759	15.72	0.655	2.733	0.01925	16	0.3849	29.22	0.8678	162.6	12.661
G20.17	18.22	0.147	4.511	0.04006	17.72	0.2003	2.556	0.04006	17.56	0.4444	33.56	0.8012	176.6	1.503
G20.18	15.39	0.294	4.822	0.12814	15.11	0.4339	2.711	0.02222	15.78	0.4444	34.11	0.8678	171.5	2.976
G20.19	16	0.2887	4.622	0.04843	15.11	0.2003	2.711	0.0294	17.56	0.4444	37.44	1.0599	158.1	6.618
G20.20	14.22	0.2422	4.567	0.07698	13.5	0.1925	2.567	0.03849	15.33	0.6667	29.89	0.5879	145.4	6.268
G20.21	15.33	0.1667	4.411	0.06759	14.39	0.147	2.444	0.04006	16.67	0.3849	33.11	0.5879	144.9	5.821
G21.01	17.11	0.2003	4.611	0.05556	16.22	0.0556	2.567	0.03849	15.78	0.5879	31.22	1.1111	174.2	7.584
G21.02	17.78	0.3643	4.533	0.1	16.94	0.4547	2.422	0.08012	18.89	0.2222	36	1.0715	176	13.19
G21.03	16.61	0.4938	4.633	0.08389	16.17	0.5092	2.556	0.09686	16	0.6667	33	1.0715	163.1	10.06
G21.04	17.5	0.0962	4.444	0.04006	17	0.0962	2.433	0.05092	18	0.3849	34.33	1.0184	154.4	4.51
G21.05	17.17	0.4194	4.533	0.05092	16.5	0.5	2.6	0.03333	16.89	0.2222	32.22	1.1277	154.8	11.692
G21.06	17.44	0.3889	4.6	0.09623	16.5	0.3333	2.567	0.06667	18	0.6667	35.11	0.5879	176.9	16.123
G21.07	15.11	0.3379	4.6	0.08389	14.44	0.3889	2.556	0.05879	15.11	0.4444	32.89	0.5879	153.3	5.36
G21.08	16.5	0.2546	4.533	0	15.67	0.2546	2.378	0.04843	18	1.0184	36.78	1.1277	162.8	5.437
G21.09	16.72	0.2422	4.778	0.06186	16	0.0962	2.733	0.07698	15.56	0.2222	30.33	0.3333	175	10.182
G21.10	17.22	0.0556	4.789	0.05879	16.61	0.1111	2.689	0.08678	17.78	0.4444	32.33	0.6939	182.4	4.029
G21.11	15.89	0.2003	4.356	0.09876	15.11	0.4006	2.344	0.02222	13.33	0.3849	31.89	0.6186	149.7	0.929
G21.12	16.83	0.1667	4.3	0.10184	15.89	0.294	2.311	0.0294	14.67	0.3849	33.56	1.1277	154.8	6.91
G21.13	17.67	0.2887	4.244	0.07286	17	0.4811	2.411	0.05879	14	0	32.67	1.1706	156.3	7.893
G21.14	17.78	0.0556	4.322	0.06759	17.17	0.0962	2.311	0.09686	16.22	0.2222	34.78	0.7286	163.9	4.197
G21.15	17.39	0.3379	4.611	0.06186	17.28	0.2003	2.578	0.04006	16	0.3849	36.78	0.8012	166.7	5.287
G21.16	17.61	0.2003	4.656	0.05879	16.72	0.1111	2.422	0.10599	18.22	0.2222	36.56	0.5879	174.2	3.067
G21.17	17.89	0.147	4.844	0.05879	17.06	0.2003	2.833	0.05774	16.67	0	34.67	0.5092	192.9	8.94
G21.18	17.78	0.8012	4.589	0.13517	17	0.866	2.644	0.07778	19.11	0.9686	31.67	1.8559	158.2	18.206
G21.19	17.61	0.4006	4.556	0.04006	17.39	0.2003	2.356	0.0294	16	0	34.22	0.6186	186.5	4.289
G21.20	18.06	0.1111	4.467	0.01925	17.33	0.0962	2.356	0.04843	17.78	0.4444	36.89	0.9095	171.1	2.96
G21.21	18.28	0.53	4.711	0.04843	17.67	0.631	2.544	0.09095	16	0.6667	37.89	1.31	197.7	9.434
G22.01	17.17	0.6667	4.511	0.04444	16.39	0.6961	2.544	0.04006	17.56	0.2222	34.44	2.5628	159.5	12.669
G22.02	16.39	0.5638	4.733	0.03333	15.67	0.6009	2.989	0.04843	17.56	0.2222	32.22	0.9686	153.8	4.15
G22.03	15.56	0.1111	4.7	0.11547	14.94	0.147	2.767	0.05774	20.67	0.3849	33.44	0.6186	148.6	5.278
G22.04	17.72	0.3379	4.5	0.05774	16.94	0.4006	2.456	0.04444	15.11	0.5879	35.89	1.1759	171.3	6.544
G22.05	17.94	0.8012	4.278	0.07286	17.44	0.7718	2.433	0.03849	17.33	0.7698	33.67	1.2019	142.6	11.438
G22.06	16.39	0.147	4.611	0.0294	15.56	0.147	2.6	0.03333	14.44	0.2222	30.67	0.5774	166.5	6.022
G22.07	16.89	0.147	4.5	0.05092	16.28	0.147	2.556	0.04843	16.22	0.4444	33.67	0.3333	159.9	5.765
G22.08	17.61	0.3889	4.5	0.2	17.06	0.3093	2.711	0.02222	15.56	0.2222	32.56	0.6186	176.7	7.671
G22.09	17.78	0.3093	4.533	0.15031	17.11	0.3889	2.722	0.04006	18.44	0.4444	35.11	0.5879	171.9	2.109
G22.10	17.44	0.0556	4.4	0.08819	16.89	0.1111	2.344	0.04006	13.33	0.7698	34.56	0.4006	157.3	5.11
G22.11	18.39	0.6961	4.456	0.15556	17.72	0.5472	2.456	0.02222	16.22	0.8012	33.67	1.8359	153.3	19.239
G22.12	16.06	0.2003	4.433	0.09623	15.5	0.0962	2.533	0.03333	14	0.3849	33.67	0.3849	152.9	8.063
G22.14	17.94	0.4006	4.5	0.01925	17.44	0.3093	2.322	0.01111	15.11	0.4444	35.44	0.5556	168	6.039
G22.15	18.78	0.2778	4.289	0.02222	18.06	0.147	2.322	0.0294	16.44	0.2222	38.22	0.6759	159.6	1.433
G22.16	17.17	0.1667	4.156	0.20031	16.33	0.1667	2.367	0.13472	14.22	0.2222	32.67	0.5092	154.3	4.159
G22.17	17.61	0.4444	4.3	0.05092	16.61	0.2003	2.156	0.02222	15.33	0	32.89	1.7462	161.6	5.687
G22.18	17.33	0.4194	4.7	0.06667	16.61	0.3643	2.944	0.09686	16.67	0.3849	31.56	0.7778	174	7.211
G22.19	18.5	0.2546	4.844	0.02222	17.89	0.2778	2.8	0.01925	18.44	0.2222	37.11	0.1111	196.5	6.026
G22.20	17.61	0.2778	4.578	0.0294	16.78	0.2422	2.589	0.02222	14.67	0.3849	30.78	0.5879	161.6	1.972
G22.21	18.89	0.5879	4.589	0.05879	18.33	0.5853	2.589	0.04006	17.11	0.2222	33.67	1.3472	177.9	5.904
G23.01	18.94	0.1111	4.744	0.04444	18.39	0.147	2.711	0.06186	14.44	0.2222	34.22	0.2222	202	3.307

G23.02	19.28	0.0556	4.756	0.04444	18.61	0.0556	2.633	0.03333	17.78	0.2222	36.33	0.8389	201.4	3.943
G23.03	15.89	0.2778	4.467	0.06939	15.11	0.147	2.544	0.01111	15.11	0.4444	34	0.8819	146	5.723
G23.04	17.39	0.3643	4.467	0.15031	16.61	0.3379	2.522	0.0294	19.11	1.2373	34.67	0.5092	147.1	17.526
G23.05	16.83	0.4194	4.3	0.11706	16	0.3469	2.333	0.08389	14	0.7698	34.22	0.9493	142.5	12.096
G23.06	17.5	0.4194	4.233	0.06939	16.61	0.4339	2.211	0.02222	14.89	0.4444	36.11	1.2814	140	7.011
G23.07	18.33	0.6667	4.478	0.07286	17.61	0.5638	2.567	0.01925	14.44	0.2222	35.11	1.0943	175.6	11.882
G23.08	18.78	0.3093	4.322	0.01111	17.83	0.2546	2.344	0.04843	15.78	0.2222	35.33	1.0715	156.5	5.052
G23.09	18.72	0.3643	4.256	0.09095	17.92	0.433	2.256	0.01111	15.33	0.7698	36.67	0.8389	167.8	7.829
G23.10	18.72	0.1111	4.389	0.04444	17.83	0.2546	2.289	0.04843	15.56	0.8012	38.67	0.7698	177	5.93
G23.11	17.39	0.3889	4.422	0.06186	16.78	0.3889	2.422	0.0294	13.78	0.5879	35.33	1.0184	157.1	5.166
G23.12	18.06	0.8296	4.256	0.09493	17.28	0.7778	2.356	0.0294	15.33	0.3849	37.11	2.2222	147.6	21.138
G23.13	18.67	0.5774	4.456	0.04843	18.06	0.4843	2.567	0.05774	14	0.3849	35.56	0.1111	164.4	7.983
G23.14	18.61	0.5472	4.278	0.07778	18.28	0.4938	2.4	0.05092	15.56	0.2222	36.22	0.8012	157	6.954
G23.15	17.89	0.0556	4.367	0.06939	17.5	0.0962	2.522	0.0294	14	0	34.89	0.6759	145.9	5.583
G23.16	18.06	0.53	4.278	0.09686	17.33	0.441	2.444	0.04843	15.56	0.4444	35.56	0.2222	137.5	13.496
G23.17	16.44	0.0556	4.689	0.08678	15.72	0.0556	2.622	0.0294	15.33	0.6667	34.78	0.2222	168.8	7.227
G23.18	17.5	0.1667	4.667	0.06667	16.72	0.0556	2.6	0.01925	18	0	37.44	0.5879	172.8	1.051
G23.19	17.06	0.2003	4.722	0.08012	16.17	0.0962	2.689	0.02222	15.78	0.4444	36	0.3333	161.5	7.622
G23.20	17.39	0.4006	4.644	0.07286	16.33	0.3469	2.656	0.06759	18	0.3849	34.56	1.0943	160.1	16.83
G23.21	16.28	0.4747	4.5	0.03333	15.67	0.2887	2.478	0.06759	14.22	0.5879	31.56	0.4843	148.2	6.085
G24.01	17.22	0.2422	4.389	0.06186	16.33	0.1925	2.511	0.0294	14.67	0.6667	32.89	0.4444	150.4	5.937
G24.02	16.33	0.1667	4.211	0.09686	15.56	0.147	2.456	0.04444	12.44	0.5879	33	0.5092	132.8	9.08
G24.03	17.83	0.0962	4.344	0.06186	16.89	0.1111	2.389	0.01111	15.11	0.5879	37	0.5092	160.4	4.413
G24.04	17.94	0.2003	4.389	0.04444	17.06	0.2003	2.589	0.02222	13.56	0.2222	31.78	0.6186	150.2	4.923
G24.05	17.89	0.2003	4.567	0.01925	17.22	0.294	2.567	0.01925	16.89	0.2222	33.44	0.4444	163.3	1.502
**$LSD_{0.05}$**	**1.1**		**0.21**		**1.08**		**0.13**		**1.35**		**2.66**		**24.37**	

Table S2. Average values of the observed traits for four individual hybrids.

Hybrid	Weight of one thousand grain [g]		Yield from the plot [kg]		dry matter content after harvest [t ha^{-1}]		Yield [t ha^{-1}]	
	Mean	s.e.	Mean	s.e.	Mean	s.e.	Mean	s.e.
G15.18	252.3	14.89	5.527	0.3067	82.42	0.3915	10.76	0.5581
G15.19	363.3	14.59	8.49	0.1332	78.74	0.1642	15.79	0.2684
G15.20	340.5	11.79	8.917	0.0788	79.88	0.1901	16.83	0.1819
G15.21	280	23.56	6.863	0.1707	81.95	0.3786	13.29	0.3641
G16.01	244.6	15.59	6.687	0.2685	82.59	0.3415	13.04	0.5114
G16.02	333.1	16.07	7.53	0.468	80.03	0.2262	14.24	0.9246
G16.03	270.1	11.92	7.837	0.1359	81.8	0.1462	15.15	0.2826
G16.04	346.2	16.62	8.733	0.0906	77.65	0.0291	16.02	0.1706
G16.05	283.3	4.77	8.313	0.1594	80.17	0.2368	15.74	0.2562
G16.06	332.9	13.49	7.003	0.2892	79.85	0.251	13.21	0.5587
G16.07	272.3	13.26	7.313	0.1415	82.32	0.245	14.22	0.2883
G16.08	326.4	10.12	7.257	0.384	81.65	0.047	14	0.7479
G16.09	272	12.75	7.61	0.3758	82.31	0.1937	14.8	0.7303
G16.10	321.3	9.03	7.783	0.3417	79.22	0.0578	14.57	0.6349
G16.11	283.4	4.4	8.33	0.1323	81.48	0.1244	16.03	0.2533
G16.12	347.1	6.49	7.993	0.2842	80.17	0.4458	15.14	0.4846
G16.13	258.4	17.89	8.353	0.197	80.03	0.2643	15.79	0.3227
G16.14	352.9	11.83	8.397	0.1485	78.81	0.6157	15.64	0.3409
G16.15	273.4	9.67	8.6	0.4139	80.43	0.0917	16.34	0.79
G16.16	328.6	14	8.143	0.4319	77.5	0.2938	14.91	0.7828
G16.17	286.3	5.64	8.227	0.0617	79.72	0.1054	15.5	0.1126
G16.18	350.7	19.14	8.053	0.0318	78.56	0.3094	14.95	0.0777
G16.19	281	3.88	7.913	0.2161	80.34	0.135	15.02	0.3941
G16.20	344.4	7.22	7.153	0.1027	77.35	0.3523	13.07	0.2058
G16.21	294.2	7.79	8.453	0.202	78.14	0.0956	15.6	0.3588
G17.01	351.3	42.24	7.46	0.1662	79.82	0.5317	14.07	0.3407
G17.02	299.3	10.92	7.12	0.4015	80.49	0.0709	13.54	0.7541
G17.04	333.3	10.66	9.063	0.3937	79.86	0.5183	17.1	0.826
G17.05	386.3	12.16	8.44	0.07	78.57	0.4136	15.67	0.1559
G17.06	319.9	4.93	7.66	0.1457	80.07	0.5127	14.49	0.3659
G17.07	341.3	7.41	7.343	0.4206	80.08	0.5015	13.88	0.7568
G17.08	284.1	8.89	7.31	0.335	81.42	0.2154	14.07	0.6817
G17.09	329	5.02	7.533	0.4004	78.87	0.319	14.03	0.7048
G17.10	266.6	8.72	7.473	0.1325	81.54	0.2979	14.4	0.2959
G17.11	275.4	18.58	6.987	0.205	80.5	0.5044	13.29	0.4486
G17.12	234.8	8.55	6.987	0.3762	82.03	0.4225	13.55	0.7938
G17.13	311.2	15.97	8.043	0.4874	81.55	0.0384	15.5	0.9462
G17.14	229.2	6.06	6.8	0.2466	81.86	0.6631	13.15	0.533
G17.15	342.1	5.21	7.227	0.461	81.9	0.1323	13.98	0.8695
G17.16	275.4	6.45	6.96	0.1484	82.99	0.1562	13.64	0.2667
G17.17	306.6	11.78	7.623	0.2368	78.58	0.6154	14.15	0.3355
G17.18	254.3	14.71	6.993	0.1521	81.43	0.3115	13.45	0.3372
G17.19	364.3	5.53	7.757	0.532	78.64	0.1501	14.41	0.9868
G17.20	283.6	5.34	6.97	0.5776	80.6	0.4423	13.26	1.0232
G17.21	371.7	23.77	8.32	0.2307	78.33	0.4868	15.39	0.3372
G18.01	269.3	4.14	7.49	0.408	79.67	0.5989	14.11	0.8576
G18.02	356.4	2.33	7.453	0.302	79.07	0.8317	13.91	0.4576
G18.03	274.6	23.6	6.517	0.5621	81	0.0917	12.47	1.0766

G18.04	321.4	11.74	8.06	0.2888	79.84	0.361	15.2	0.4767
G18.05	288.9	3.22	7.75	0.1457	81.62	0.4612	14.94	0.2252
G18.06	379.2	22.3	6.953	0.341	78.22	0.2854	12.85	0.5889
G18.07	322.4	11.5	7.433	0.1974	80.43	0.1343	14.12	0.3973
G18.08	298.1	4.34	7.107	0.1633	80.74	0.11	13.56	0.325
G18.09	241.8	5.95	7.96	0.3653	81.2	0.1357	15.27	0.7218
G18.10	329.3	12.18	8.113	0.2221	80.23	0.3583	15.38	0.3635
G18.11	267.3	15.9	7.423	0.4186	81.73	0.3374	14.33	0.7475
G18.12	380.8	0.53	8.07	0.38	78.36	0.4843	14.93	0.6143
G18.13	313.3	4.06	7.653	0.133	81.9	0.0338	14.81	0.2513
G18.14	370.3	4.89	7.96	0.085	79.28	0.4452	14.91	0.0757
G18.15	297.3	3.62	7.953	0.0669	81.05	0.3325	15.23	0.1908
G18.16	320	13.99	7.317	0.2463	80.3	0.2318	13.88	0.4734
G18.17	240.4	6.97	7.047	0.104	80.9	0.1304	13.47	0.2161
G18.18	376.8	21.6	8.127	0.3217	80.02	0.283	15.36	0.6127
G18.19	292	6.95	7.597	0.0754	82.05	0.1601	14.73	0.1738
G18.20	370.6	35.73	6.487	0.3121	77.32	0.8467	11.86	0.6664
G18.21	334.4	14.4	7.053	0.5154	80.84	0.4234	13.47	0.9571
G19.01	355.8	14.85	7.563	0.5906	81.26	0.4992	14.52	1.1542
G19.02	283.4	7.97	7.673	0.4684	82.27	0.3436	14.92	0.9704
G19.03	319.2	11.33	7.463	0.1538	79.93	0.3094	14.1	0.3295
G19.04	301.8	15.53	7.697	0.2596	79.88	0.5164	14.52	0.4689
G19.05	335.3	23.61	6.537	0.2035	82.35	0.0953	12.71	0.3923
G19.06	304	22.58	6.513	0.2969	84.06	0.3503	12.94	0.6372
G19.07	356.8	27.54	7.967	0.4455	81.81	0.5904	15.4	0.9245
G19.08	294.5	17.69	7.54	0.1513	83.23	0.2397	14.83	0.2957
G19.09	400.4	20.33	7.657	0.4518	79.08	0.3327	14.31	0.8958
G19.10	324.8	17.91	8.133	0.3941	80.72	0.3868	15.51	0.718
G19.11	375.6	21.16	7.983	0.455	82.07	0.151	15.48	0.8645
G19.12	356.5	15.64	7.703	0.2429	83.4	0.3977	15.18	0.5237
G19.13	396.4	15.79	7.163	0.3862	80.42	0.1686	13.61	0.764
G19.14	335.1	24.38	7.68	0.2862	82.53	0.358	14.97	0.4943
G19.15	353.6	20.72	6.98	0.4	80.8	0.4504	13.32	0.7557
G19.16	304.2	25.32	7.053	0.3139	83.38	0.1986	13.89	0.6006
G19.17	384.6	43.07	8.123	0.3604	79.5	0.1808	15.26	0.6831
G19.18	328.3	8.72	7.443	0.3693	81.14	0.3056	14.26	0.6624
G19.19	393.8	12.48	8.287	0.2972	79.09	0.0721	15.48	0.5411
G19.20	339.9	5.43	7.993	0.2541	81.77	0.247	15.44	0.5384
G19.21	304.7	16.87	7.92	0.3482	81.69	0.2187	15.28	0.6571
G20.01	258.7	16.62	8.363	0.0491	81.7	0.4028	16.14	0.133
G20.02	311.3	11.56	7.087	0.2146	80.85	0.12	13.53	0.4286
G20.03	228.3	9.81	7.413	0.3384	82.05	0.0233	14.37	0.6531
G20.05	288	8.17	7.39	0.2689	81.07	0.1217	14.15	0.4979
G20.06	346.4	12.34	7.377	0.6671	78.72	0.3957	13.72	1.2552
G20.07	288.2	5.33	7.743	0.1965	80.37	0.012	14.7	0.3745
G20.09	290.1	14.69	6.667	0.1525	82.64	0.3996	13.01	0.2976
G20.10	345.2	5.28	8.147	0.0784	80.92	0.145	15.57	0.1378
G20.11	306.4	1.82	7.737	0.0267	82.44	0.282	15.07	0.0533
G20.12	292.1	16.36	6.93	0.0436	81.29	0.4431	13.31	0.1097
G20.13	264.5	11.17	7.07	0.3233	82.44	0.167	13.77	0.6034
G20.14	307.4	18.16	6.537	0.1637	81.52	0.1212	12.59	0.2987
G20.15	308.4	46.22	6.567	0.3937	82.94	0.2484	12.87	0.8016
G20.16	347.9	25.17	8.44	0.2542	77.47	0.1517	15.45	0.4879

G20.17	300.8	14.82	8.443	0.2315	80.1	0.3434	15.97	0.3753
G20.18	319.3	10.44	7.6	0.1436	78.43	0.3121	14.08	0.3215
G20.19	240.6	6.39	7.123	0.4191	81.22	0.5505	13.66	0.706
G20.20	317.6	7.43	6.207	0.2463	80.66	0.1729	11.83	0.4933
G20.21	262.4	3.12	7.197	0.12	83.13	0.1082	14.13	0.2284
G21.01	354.3	10.09	8.483	0.177	77.66	0.2457	15.57	0.3494
G21.02	258.4	13.47	7.647	0.3889	81.06	0.5995	14.65	0.8479
G21.03	308.9	9.62	6.497	0.1445	82.38	0.1753	12.65	0.3061
G21.04	250.3	9.55	6.377	0.2769	83.79	0.4237	12.63	0.5995
G21.05	283.7	11.2	7.487	0.2273	78.9	0.467	13.95	0.4083
G21.06	278.4	11.48	7.077	0.2804	81.79	0.0376	13.67	0.5418
G21.07	308.4	4.2	6.82	0.3485	81.41	0.257	13.11	0.6277
G21.08	248	18.54	7.417	0.1424	81.97	0.2439	14.36	0.2985
G21.09	370.7	19.83	8.203	0.5324	78.63	0.1748	15.24	0.9726
G21.10	317.8	7.44	7.987	0.2892	81.45	0.2022	15.36	0.5179
G21.11	352.6	7.8	8.117	0.4504	80.92	0.1124	15.52	0.8813
G21.12	314.8	11.4	7.233	0.1543	82.55	0.2931	14.11	0.3227
G21.13	341.4	6.19	6.087	0.2439	82.64	0.2686	11.88	0.4924
G21.14	290.6	3.43	6.857	0.3645	83.69	0.351	13.56	0.7594
G21.15	283.4	4.45	5.357	0.3567	80.86	0.2274	10.23	0.6745
G21.16	261.5	2.57	6.613	0.3358	81.7	0.1212	12.76	0.6631
G21.17	333.6	12.22	8.547	0.3134	79.59	0.3148	16.07	0.5782
G21.18	259.8	3.11	8.037	0.4836	82.05	0.5015	15.58	0.9617
G21.19	340.6	6.38	7.807	0.0857	80.13	0.2085	14.78	0.1723
G21.20	261.2	2.55	8.137	0.3169	82.23	0.4398	15.8	0.5646
G21.21	327	15.5	6.573	0.4561	80.28	0.2761	12.46	0.8249
G22.01	263.5	1.13	6.94	0.5587	81.98	0.1562	13.44	1.0621
G22.02	272.5	11.84	7.27	0.6412	81.76	0.4133	14.05	1.2928
G22.03	215.8	14.36	6.707	0.582	82.44	0.3091	13.06	1.1333
G22.04	316.5	8.61	7.9	0.3051	80.86	0.3696	15.09	0.5613
G22.05	243.9	7.33	7.043	0.1954	82.99	0.3875	13.81	0.4116
G22.06	375.7	4.6	8.133	0.0333	79.68	0.2433	15.31	0.0956
G22.07	292.9	8.01	7.707	0.1398	81	0.447	14.75	0.347
G22.08	348.5	5.95	7.37	0.0569	80.96	0.3492	14.1	0.1241
G22.09	265.7	3.02	7.55	0.2203	82.16	0.5541	14.65	0.3694
G22.10	342.2	5.3	6.59	0.1436	82.92	0.1703	12.91	0.2669
G22.11	277.9	10.29	7	0.1358	82.58	0.0617	13.65	0.2735
G22.12	326.2	27.22	7.487	0.1676	81.63	0.0493	14.44	0.3212
G22.14	313.6	3.43	6.137	0.318	83.28	0.3581	12.07	0.5759
G22.15	254.1	6	7.04	0.4701	84.42	0.2541	14.04	0.965
G22.16	332.1	2.94	7.053	0.2925	81.25	0.3329	13.54	0.6141
G22.17	321.2	9	6.963	0.3317	83.1	0.3765	13.67	0.6847
G22.18	331.3	14.41	8.163	0.3645	80.09	0.1517	15.44	0.6678
G22.19	287.1	7.99	8.137	0.0845	80.81	0.1858	15.54	0.1821
G22.20	358.6	8.71	7.5	0.31	79.75	0.3407	14.13	0.6346
G22.21	309.1	4.25	7.53	0.2695	80.15	0.259	14.26	0.5312
G23.01	408.7	3.61	8.827	0.2251	80.4	0.1884	16.76	0.4027
G23.02	312	5.86	8.353	0.3643	81.65	0.2425	16.12	0.7505
G23.03	285	14.76	6.983	0.1988	81.24	0.2136	13.41	0.4101
G23.04	220.1	10.75	6.53	0.2113	83.06	0.1291	12.81	0.4015
G23.05	296.4	8.53	6.667	0.3797	82.06	0.3279	12.92	0.7238
G23.06	261.2	15.13	6.373	0.1812	84.22	0.3587	12.68	0.365
G23.07	345.2	8.26	8.21	0.5009	80.77	0.2463	15.67	0.9712

G23.08	280.8	4.7	7.683	0.1313	82.02	0.2826	14.89	0.2937
G23.09	298.7	3.47	7.91	0.1947	76.36	0.4318	14.27	0.3053
G23.10	295.7	16.07	7.497	0.3538	82.6	0.2998	14.63	0.6434
G23.11	323.6	10.2	6.9	0.4535	82.46	0.2331	13.44	0.8498
G23.12	258.1	28.86	6.117	0.3768	83.88	0.1852	12.12	0.7483
G23.13	330.2	10.94	6.72	0.4331	82.29	0.1422	13.07	0.8574
G23.14	278.8	10.84	5.923	0.4048	82.53	0.0874	11.55	0.7973
G23.15	298.9	13.4	6.107	0.4077	82.82	0.2467	11.94	0.7679
G23.16	247.5	16.96	6.21	0.2875	83.56	0.0984	12.26	0.5701
G23.17	316.7	4.01	8.46	0.1901	79.77	0.2539	15.94	0.4077
G23.18	256.6	5.32	8.103	0.167	82.01	0.3171	15.69	0.2887
G23.19	285	16.5	7.243	0.4288	81.85	0.563	14	0.7302
G23.20	256	17.36	7.807	0.2949	81.76	0.4682	15.08	0.5671
G23.21	330.5	8.42	7.413	0.2033	80.1	0.5228	14.02	0.3067
G24.01	311.9	1.81	7.08	0.3118	81.2	0.3634	13.59	0.6584
G24.02	322.9	10.76	6.64	0.1825	82.14	0.112	12.89	0.3583
G24.03	287.5	10.33	7.283	0.1641	82.67	0.4446	14.22	0.3468
G24.04	348.5	2.35	6.82	0.2574	81.77	0.3667	13.17	0.477
G24.05	289.4	6.34	7.503	0.2693	83.14	0.2693	14.74	0.5787
**$LSD_{0.05}$**	**38.84**		**0.868**		**0.92**		**1.666**	
